# Progress in Catalytic Asymmetric Reactions with 7-Azaindoline as the Directing Group

**DOI:** 10.3390/molecules28237898

**Published:** 2023-12-01

**Authors:** Yan-Ping Zhang, Yong You, Jun-Qing Yin, Zhen-Hua Wang, Jian-Qiang Zhao, Wei-Cheng Yuan

**Affiliations:** 1 School of Pharmacy, Chengdu University, Chengdu 610106, China; zhangyanping@cdu.edu.cn; 2 Institute of Advanced Study, Chengdu University, Chengdu 610106, China; yinjunqing@cdu.edu.cn (J.-Q.Y.);

**Keywords:** 7-azaindoline amides, directing group, asymmetric synthesis, enolization chemistry, metal catalysis, organocatalysis

## Abstract

α-Substituted-7-azaindoline amides and α,β-unsaturated 7-azaindoline amides have emerged as new versatile synthons for various metal-catalyzed and organic-catalyzed asymmetric reactions, which have attracted much attention from chemists. In this review, the progress of research on 7-azaindoline amides in the asymmetric aldol reaction, the Mannich reaction, the conjugate addition, the 1,3-dipole cycloaddition, the Michael/aldol cascade reaction, aminomethylation and the Michael addition-initiated ring-closure reaction is discussed. The α-substituted-7-azaindoline amides, as nucleophiles, are classified according to the type of α-substituted group, whereas the α,β-unsaturated 7-azaindoline amides, as electrophiles, are classified according to the type of reaction.

## 1. Introduction

Carboxylic acids are widely distributed in nature and are compounds that occur naturally at various stages of the life cycle, such as the living-organism–Krebs cycle, fermentation processes, and geological processes. Carboxylic acids are often present as a functional group in effective medicines, and approximately a quarter of all commercialized pharmaceuticals contain a carboxylic acid group [1,2]. In addition, carboxylic acids play important roles in the food industry, like ascorbic acid, which functions as an antioxidant, propionic acid, which functions as a flavor, and lactic acid, which functions as a preservative [3,4,5,6,7,8]. Accordingly, the synthesis and functionalization of carboxylic acids are of great importance in the development of functional materials, pharmaceuticals, and various other fields [9,10,11,12,13,14]. Because of the resonance of the carboxyl proton between its two oxygen atoms, the carboxyl group usually exhibits a low reactivity, making it uncommon for carboxylic acids to directly participate in reactions [14]. For example, it is difficult for α,β-unsaturated carboxylic acids to undergo the Michael addition, which often readily occurs for α,β-unsaturated carbonyl compounds [15,16,17,18,19,20,21]. Therefore, exploitation of the surrogates of carboxylic acid is, and will continue to be, highly desirable. In this context, considerable efforts have been made by chemists and substantial progress has been made. For instance, esters are important substrates for the following reactions [22,23,24,25,26,27]: (1) Aron et al. [22] reported in 2010 that Ca(OTf)_2_-catalyzed *α,β*-unsaturated esters participate in a [3 + 2] cycloaddition reaction with unprotected amino acid esters and aldehydes, resulting in the formation of polysubstituted pyrrolidines; (2) Shang et al. [23] developed the Michael addition reaction of 1-oxoindane-2-carboxylic acid esters with *β*-ester enone, catalyzed by an organophosphine containing multiple hydrogen-bond donors. Nevertheless, low substrate activity and selectivity remain issues in the development of carboxylic acid surrogates.

Directing groups have been used to enhance the reactivity and regulate the stereoselectivity of reactions in asymmetric transformations [28]. If the substrate is not suitable for effective interactions with the catalyst or reagent to promote the selective reaction, the appropriate design of a directing group could be feasible. In general, the directing group is expected to satisfy the following requirements: (i) is easy to install in the molecular structure, (ii) ensures efficient control of reaction activity and selectivity, and (iii) is easily removed from the molecular structure. Taking this into account, the introduction of a directing group on the carboxyl group is proposed in order to enhance the reactivity and stereoselectivity of reactions involving carboxylic acids as starting materials.

**Scheme 1:**
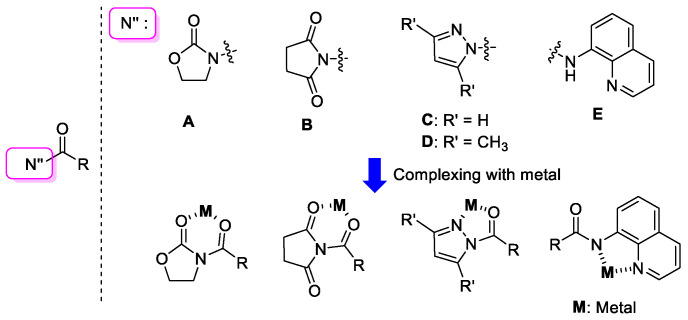
Several types of directing groups.

At present, the main types of directing groups are as follows: 2-oxazolidone (**A**) [29,30,31,32,33,34,35,36,37,38,39,40], tetrahydropyrrole 2,5-dione (**B**) [41,42,43], pyrazole or 3,5-dimethylpyrazole (**C** or **D**) [44,45,46,47,48,49,50,51,52,53,54,55,56], and 8-aminoquinoline (**E**) [57,58,59,60,61,62,63,64,65]. They coordinate with metals to form stable five- or six-membered ring complexes that control the stereoselectivity of the reaction through a specific chiral environment (Scheme 1). Despite these seminal works, some challenges remain for the functionalization of carboxylic acids, including the harsh conditions for the removal of the directing group, unsatisfactory stereocontrol, and low activity. Recently, Shibasaki’s group reported a series of asymmetric reactions involving amide substrates with 7-azaindoline as the directing group. The advantages of 7-azaindoline are as follows: (i) it improves the reaction activity and stereoselectivity; (ii) the 7-azaindoline group in the product is easily removed, facilitating the formation of carboxylic acid and carboxylic acid derivatives; (iii) the complexation of 7-azaindoline with metal reagents can prevent over-reduction, -oxidation and dehalogenation. Yuan’s group has also conducted some research using 7-azaindoline amides as substrates.

**Scheme 2:**
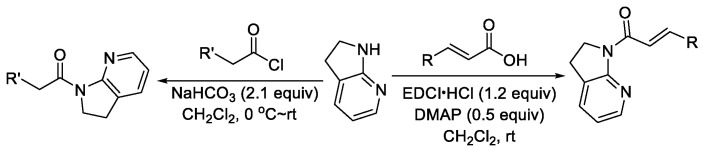
Preparation of *α*-substituted-7-azaindoline amides and *α,β*-unsaturated 7-azaindoline amides.

The 7-azaindoline amides reported in the literature are divided into two categories. One is the *α*-substituted-7-azaindoline amide, which is a carbonyl compound with weak acidity and usually acts as the nucleophile of an asymmetric direct aldol or Mannich reaction. The other is the *α,β*-unsaturated 7-azaindoline amide, which is an *α*,*β*-unsaturated carbonyl compound with poor electrophilicity and is generally regarded as an electrophile in asymmetric reactions. The preparation method of 7-azaindoline amide is as follows: *α*-substituted-7-azaindoline amides are synthesized from 7-azaindoline and *α*-substituted acetyl chloride using 1.2 equivalents of NaHCO_3_ in CH_2_Cl_2_, which have been recognized as efficient substrates. *α,β*-unsaturated 7-azaindoline amides, which were prepared from 7-azaindoline and cinnamic acids using 1.2 equivalents of 1-(3-dimethylaminopropyl)-3-ethylcarbodiimide hydrochloride (EDCI·HCl) and 0.5 equivalents of 4-dimethylaminopyridine (DMAP) in CH_2_Cl_2_, were found to be stable and highly efficient synthons (Scheme 2). In this review, we will summarize the state-of-the-art catalytic asymmetric reactions involving 7-azaindoline amides as nucleophiles and electrophiles, respectively.

## 2. α-Substituted-7-Azaindoline Amide as a Nucleophile

On the basis of the catalytic asymmetric reactions reported so far, 7-azaindoline amides can be divided into six major types: (1) *α*-sulfanyl 7-azaindoline amide, (2) fluoroalkyl 7-azaindoline amide, (3) *α*-azido 7-azaindoline amide, (4) *α*-alkyl 7-azaindoline amide, (5) *α*-halo 7-azaindoline amide, and (6) α-oxygen 7-azaindoline amide. These amides are a new class of latent enolates developed by Shibasaki and co-workers, which can react with aldehydes, ketones and imines under the catalysis of synergistic catalysts to obtain corresponding products with high yields and stereoselectivities.

### 2.1. α-Sulfanyl 7-Azaindoline Amide as an Aldol Donor

**Scheme 3:**
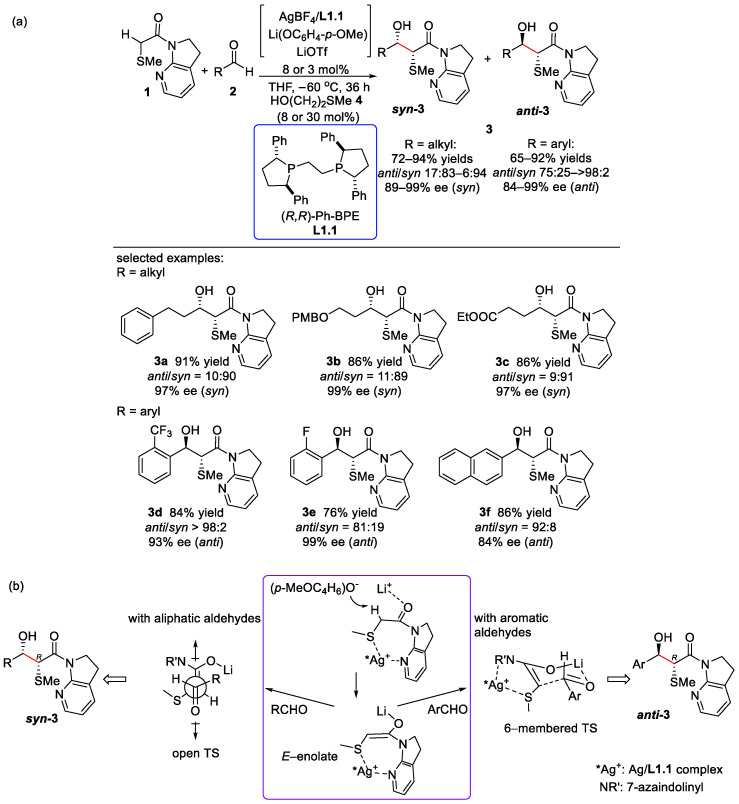
(**a**) Asymmetric aldol reaction of α-sulfanyl 7-azaindoline amide with aldehydes. (**b**) Plausible reaction mechanism of the aldol reaction.

The first example of the utilization of 7-azaindoline amide as a nucleophile in the asymmetric aldol reaction was reported by Shibasaki in 2014 [66]. In more detail, the *α*-sulfanyl 7-azaindoline amide **1** was converted to the corresponding *β*-hydroxy carbonyl compounds **3** using aliphatic or aromatic aldehydes **2** as aldol acceptors, catalyzed by the AgBF_4_/(*R*,*R*)-Ph-BPE **L1.1** complex with LiOTf and 2-methylthioethanol **4** as additives and Li(OC_6_H_4_-*p*-OMe) as a Brønsted base (Scheme 3a). The interaction of *α*-sulfanyl 7-azaindoline amide **1** with the monomer Ag(I) complex enhanced its acidity, and the proton on the methylene group of *α*-sulfanyl 7-azaindoline amide **1** is more easily removed by Li(OC_6_H_4_-*p*-Me) during the reaction. The NMR and X-ray crystallographic analysis showed that the dimeric silver complex Ag_2_(**L1.1**)_2_(OTf)_2_ was dominant in THF solution of Ag(I)/**L1.1**/LiOTf. The ESI-MS analysis revealed that *α*-sulfanyl 7-azaindoline amide **1** promoted the balance of monomeric and dimeric complexes. When aliphatic aldehydes were used as aldol acceptors, the corresponding aldol products **3** were obtained in good to high yields (72–94% yields) with high levels of stereoselectivities (16:84–6:94 dr, 89–99% ee (*syn*)). However, when aromatic aldehydes were used as the aldol acceptors, the aldol products **3** were obtained with moderate to good yields (65–92%) and high stereoselectivities (81:19→98:2 dr, 84–99% ee (*anti*)). Compared with aliphatic aldehydes, aromatic aldehydes reacted with *α*-sulfanyl 7-azaindoline amide **1** more quickly, but the retro–aldol reaction resulted in a significant decrease in enantioselectivity. Fortunately, 2-methylthioethanol **4**, as a fake, can effectively inhibit the occurrence of the retro–aldol reaction. The potential synthetic utility of the methodology in this work was demonstrated by the further conversion of product **3**.

The authors proposed a plausible reaction mechanism. The *α*-sulfanyl 7-azazindoline amide and Ag/**L1.1** complex form a seven-membered ring structure through bidentate chelation, which causes the 7-azazindoline group of the amide to tilt, partially breaking the conjugated structure. The carbonyl group of the seven-membered ring structure binds to the hard Lewis acid Li^+^, and the *α*-H of the amide is easily removed by a Brønsted base to form a Li enolate amide. The absolute configuration of *syn*-**3** and *anti*-**3**, obtained by the reaction of aliphatic aldehydes and aromatic aldehydes with the Li enolate amide, consistently shows an *R* configuration at the *α*-position. This suggests that the reaction face of the Li enolate amide remains the same, but the transition state is different. The *α*-sulfanyl 7-azazindoline amide coordinates with the Ag complex to form *E*-enolate amide, and it forms *anti*-**3** with smaller aromatic aldehydes through a six-membered ring transition state. On the other hand, larger aliphatic aldehydes lead to *syn*-**3** via an open transition state with minimal spatial repulsion (Scheme 3b).

### 2.2. Fluoroalkyl 7-Azaindoline Amides as Mannich Donors or Aldol Donors

**Scheme 4:**
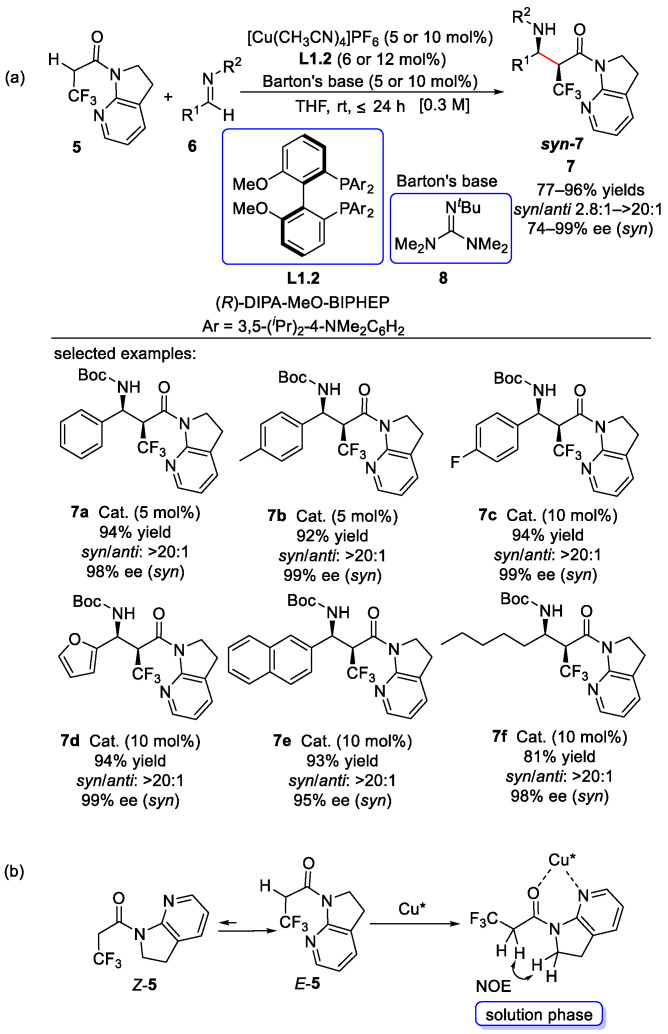
(**a**) Asymmetric Mannich reaction of α-CF_3_ 7-azaindoline amide with imines. (**b**) The (*E*)-α-CF_3_ 7-azazindoline amide was coordinated with the Cu(I) * complex.

Based on the application of *α*-sulfanyl 7-azaindoline amide in the catalytic asymmetric aldol reaction (Scheme 3), Shibasaki’s group reported the first example of an asymmetric Mannich reaction of *α*-CF_3_ 7-azaindoline acetamide **5** with *N*-Boc imine **6** using the [Cu(CH_3_CN)_4_]PF_6_/**L1.2**/Barton’s base as a cooperative catalyst in 2014 (Scheme 4a) [67]. The *α*-CF_3_ enolate was catalytically generated without undesirable fluoride elimination, and the synthesis of various *N*-Boc-protected *β*-amino acid derivatives **7** was achieved in good to high yields (77–96%) with high stereoselectivities (2.8:1→20:1 dr, and 74–99% ee). The reaction exhibits a broad substrate scope, accommodating both aromatic and heteroaromatic imines in the catalytic system. In particular, the aliphatic imines with low activity were able to participate effectively in the reaction, resulting in good results for the corresponding products.

A coordination mode for the *α*-CF_3_ 7-azaindoline amide **5** and the Cu(I) complex was also brought forward, as shown in Scheme 4b. The defluorination of *α*-CF_3_ 7-azaindoline amide **5** was effectively avoided through the complexation of the pyridinyl nitrogen with the chiral Cu(I) complex. The authors also found that *α*-CF_3_ 7-azaindoline amide **5** preferred *E*-conformation in the solution using Nuclear Overhauser Effect (NOE) analysis.

**Scheme 5:**
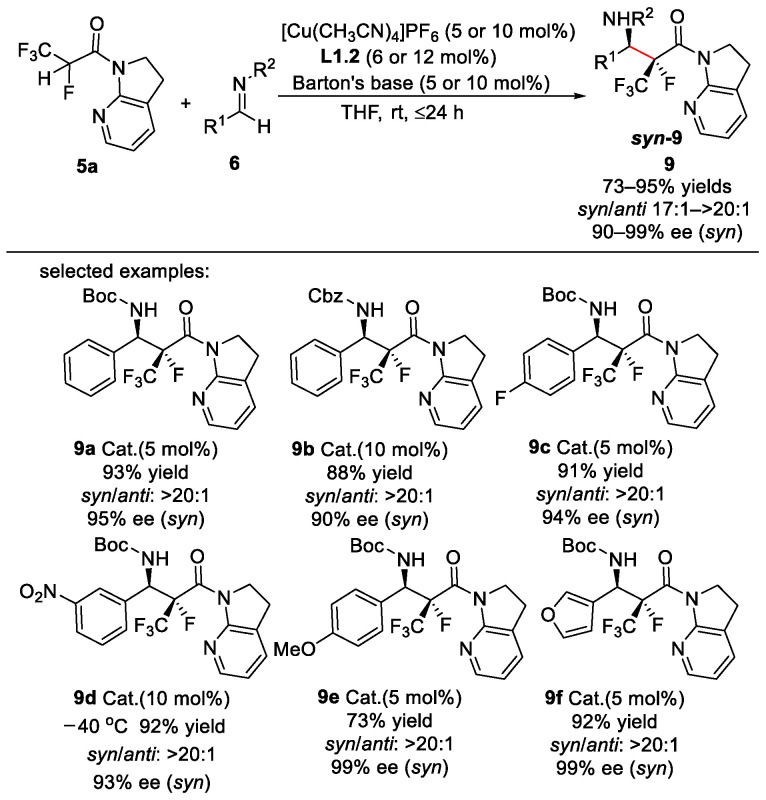
Asymmetric Mannich reaction of α-F-α-CF_3_ 7-azazindoline acetamide with imines.

**Scheme 6:**
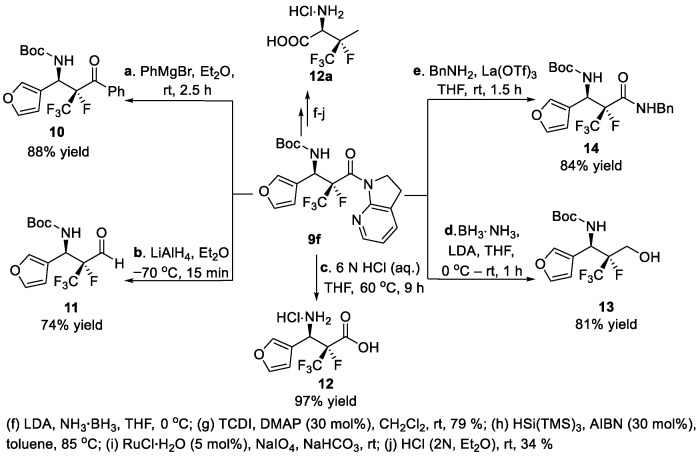
Transformations of the product **9f**.

**Scheme 7:**
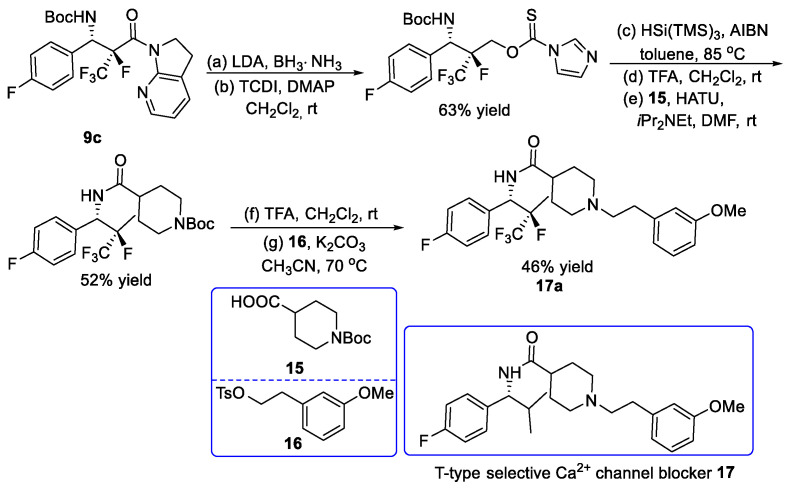
A fluorinated analogue **17a** of T-type selective Ca^2+^ channel blocker **17** was synthesized.

**Scheme 8:**
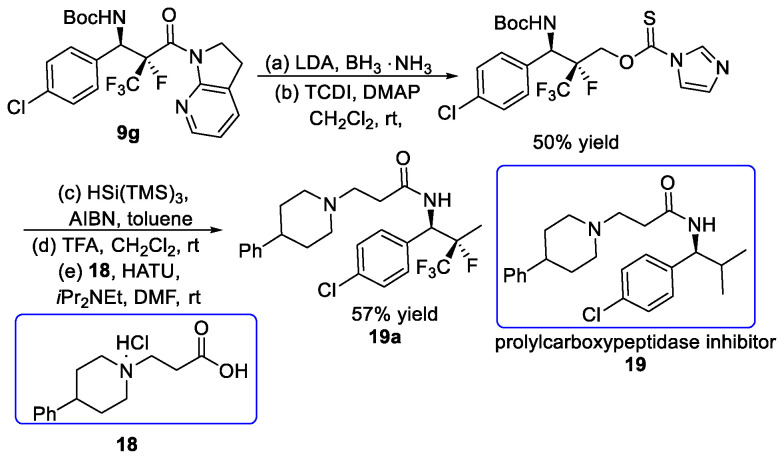
A fluorinated analogue **19a** of prolyl carboxypeptidase inhibitor **19** was synthesized.

**Scheme 9:**
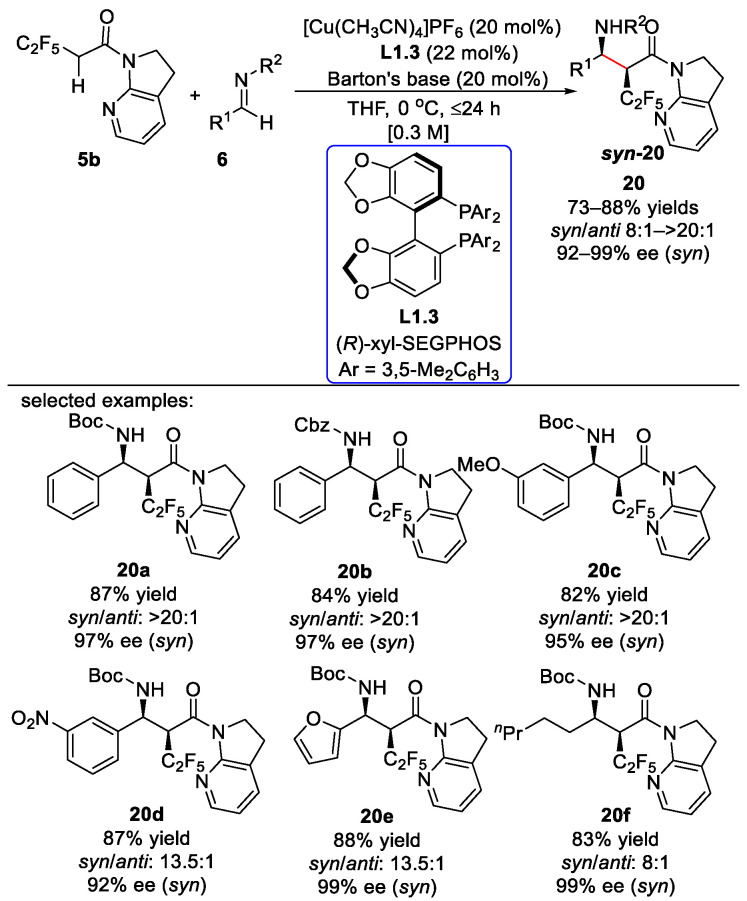
Asymmetric Mannich reaction of *α*-C_2_F_5_-7-azaindoline acetamide with imines.

**Scheme 10:**
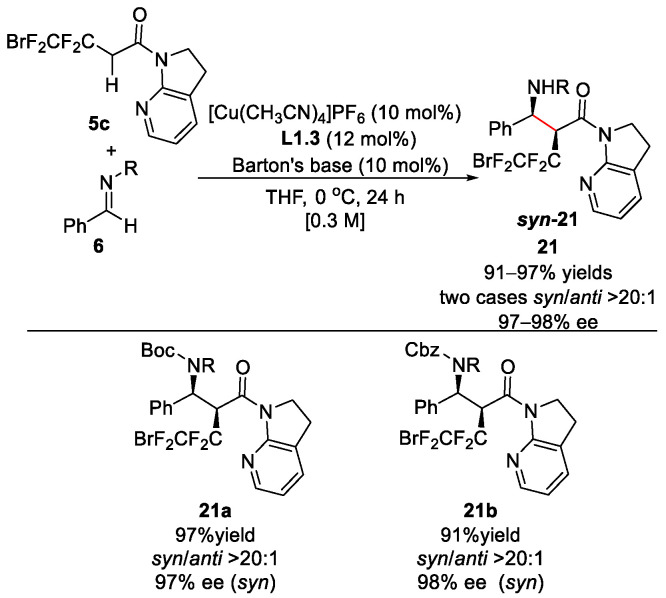
Asymmetric Mannich reaction of α-CF_2_CF_2_Br 7-azaindoline amide with imines.

**Scheme 11:**
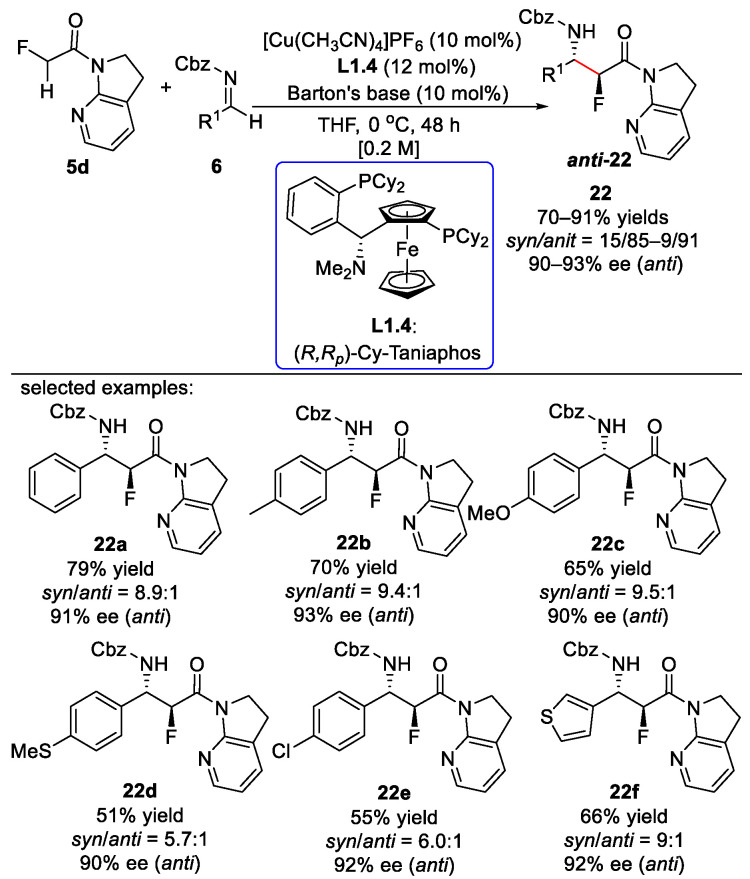
Asymmetric Mannich reaction of α-F 7-azaindoline amide with imines.

Benefiting from the investigation of the acid/base binary catalytic system, which used Barton’s base as a Brønsted base and [Cu(CH_3_CN)_4_]PF_6_ as a soft Lewis acid, Shibasaki’s group developed an exceptionally efficient method for synthesizing enantioenriched *α*-fluorinated and *α*-fluoroalkylated *β*-amino acid derivatives through the direct catalytic asymmetric Mannich reaction of other fluorinated amides **5a**–**d** with imine **6** (Scheme 5, Scheme 6, Scheme 7, Scheme 8, Scheme 9, Scheme 10 and Scheme 11). This method was developed in response to the high demand for fluorinated chiral compounds [68].

The *α*-F-*α*-CF_3_ carbonyl compounds correspond to perfluorinated analogues of propionic acid units and are common structural units of many bioactive compounds. The *α*-F-*α*-CF_3_ 7-azazindoline acetamide is very useful for constructing chiral centers containing both CF_3_ and F substitutions, which are difficult to obtain via the asymmetric fluorination or trifluoromethylation [69,70,71,72,73]. In this work, the authors constructed a family of *β*-amino acid derivatives **9** through the direct catalytic asymmetric Mannich reaction between racemic *α*-F-*α*-CF_3_ 7-azaindoline acetamide **5a** and imines **6** (Scheme 5). The *β*-amino acid derivatives **9** bearing both CF_3_ and F substituents at the stereogenic carbon center were obtained in an up to 95% yield, >20:1 dr, and 99% ee under the catalysis of [Cu(CH_3_CN)_4_]PF_6_/**L1.2**/Barton’s base. Compared to the optimization conditions determined above, the concentration of **5a** in this system was slightly increased from 0.3 M in Scheme 3 to 0.5 M in Scheme 5, which also compensated for the weaker nucleophilicity. The further conversion of product **9f** to other compounds with an unchanged chiral configuration also demonstrates the potential synthetic utility of the methodology. Notably, Product **9f** also can be converted into (2*R*,3*S*)-3,4,4,4-tetrafluorovaline hydrochloride product **12a** in 34% yield via a series of transformations [74] (Scheme 6).

More importantly, some products can be converted into biologically active substances in a few steps [75]; for example, the *β*-amino acid derivative **9c** can be converted into the fluorinated analogue **17a** of calcium channel blocker **17** via several steps (Scheme 7). Moreover, the racemic *α*-F-*α*-CF_3_ 7-azazindoline amide **5a** could also be used as a nucleophile to react with a *para*-chloro-substituted imine to synthesize the fluorinated analogue **19a** of propyl carboxypeptidase inhibitor **19** (Scheme 8). The results of the preliminary research are an important foundation for the future development of bioactive substances with an *α*-F-*α*-CF_3_ tetra-substituted stereogenic center.

To extend the range of *β*-amino acid derivatives containing fluorinated groups on the *α*-carbon, the authors also developed an asymmetric catalytic system using a less sterically hindered ligand, (*R*)-xyl-Segphos **L1.3**, in combination with [Cu(CH_3_CN)_4_]PF_6_ for the asymmetric Mannich reaction of *α*-C_2_F_5_ 7-azaindoline acetamide **5b** with amine **6**. This method generated enantiomerically enriched *α*-C_2_F_5_-*β*-amino acid derivatives **20** containing adjacent tertiary stereocenters in good yields and high enantioselectivities (Scheme 9). The electronic properties of phenyl substituents in imines have little effect on the reactivity and enantioselectivity of Mannich reactions. Finally, it was also found that aliphatic imines with low reactivity were suitable for the catalytic system.

The authors also described the asymmetric Mannich reaction of *α*-CF_2_CF_2_Br 7-azaindoline acetamide **5c** as a nucleophile with imines to synthesize a variety of *α*-CF_2_CF_2_Br *β*-amino acid derivatives **21** through the catalysis of [Cu(CH_3_CN)_4_]PF_6_/**L1.3** complex. They used plenty of imines (6 equiv) to ensure the formation of target products from the enolate, even if faster defluorination occurred during the reaction (Scheme 10). In addition, Shibasaki and co-workers also explored the catalytic asymmetric synthesis of *α*-monofluorinated *β*-amino acid derivatives **22** using a *α*-F 7-azaindoline acetamide **5d** as a nucleophile that reacted with imines (Scheme 11). In this work, *N*-Cbz imine reacted with *α*-monofluorinated amide in the presence of the [Cu(CH_3_CN)_4_]PF_6_/(*R*,*R-p*)-Cy-Taniaphos **L1.4** catalyst to provide chiral *β*-amino acid derivatives **22** bearing an *α*-F stereogenic center in high enantioselectivities. Although soft Lewis basic functional groups likely interfered with the catalysis, both the -SMe substituted imine and 3-thiophenimine reacted smoothly with **5d** in the accepted results (Scheme 11, **22d** and **22f**).

**Scheme 12:**
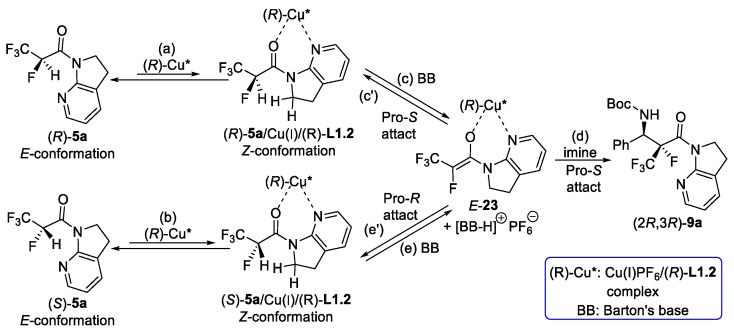
Possible pathways of the direct Mannich reaction under [Cu(CH_3_CN)_4_]PF_6_/**L1.2** complex catalysis.

Because of the inherent chirality of *α*-F-*α*-CF_3_ 7-azaindoline amide **5a**, a plausible pathway for the Mannich reaction between **5a** and an imine was proposed, as shown in Scheme 12. Firstly, both the (*R*)-amide **5a** and (*S*)-amide **5a** were combined with the chiral complex Cu(I)/(*R*)-**L1.2** (paths a, b), resulting in the formation of the **5a**/Cu(I)/(*R*)-**L1.2** complex. This complex underwent rapid reprotonation, leading to the formation of enolate *E*-**23**. Reversible enolization occurred through paths c and e. Finally, the desired adduct **9a** was generated through the irreversible Mannich reaction of enolate *E*-**23** (path d). In addition, the authors advanced the following conclusions: (i) in the presence of Barton’s base, the acidity of *α*-F-*α*-CF_3_ 7-azaindoline amide **5a** is sufficient for deprotonation, and the rate of deprotonation significantly accelerated in the presence of the Cu(I) complex; (ii) the Cu(I) complex plays a crucial role in bringing together *α*-F-*α*-CF_3_ 7-azaindoline amide **5a** and imine **6** to facilitate the formation of C-C bonds, but it does not directly accelerate deprotonation.

**Scheme 13:**
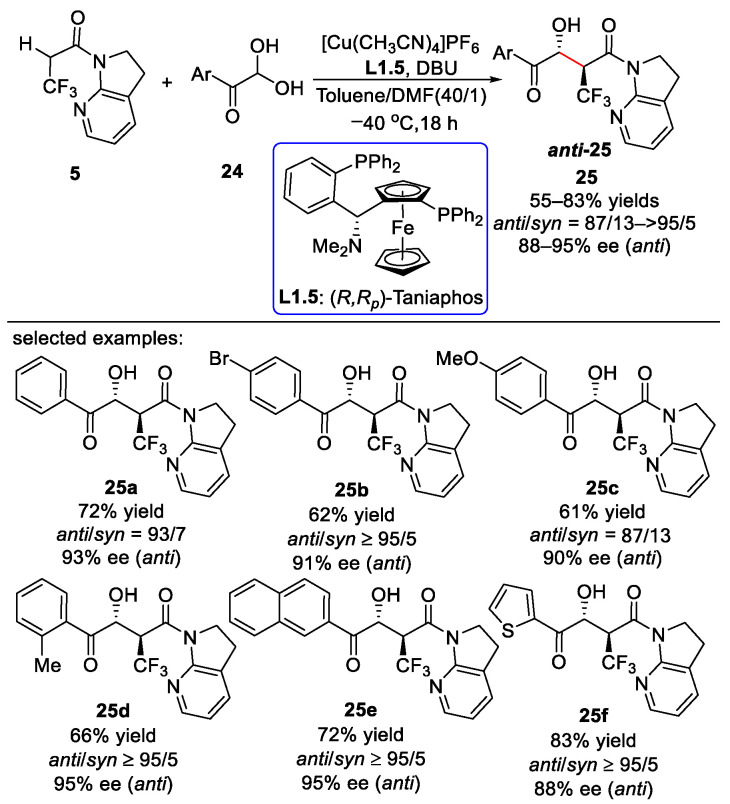
Asymmetric aldol reaction of an *α*-CF_3_ 7-azaindoline amide with arylglyoxal hydrates.

Inspired by the fact that *α*-CF_3_ 7-azaindoline acetamide was a highly efficient nucleophile for constructing chiral *α*-CF_3_ *β*-amino acid derivatives, Shibasaki and co-workers developed another class of *β*-hydroxyl compounds **25** bearing an *α*-CF_3_ stereogenic center, which were synthesized by arylglyoxal hydrates **24** and *α*-CF_3_ 7-azaindoline amide **5** via the direct catalytic asymmetric aldol reaction (Scheme 13) [76]. The catalyst was a combination of a soft Lewis acid Cu(I)/**L1.5** and a hard Brønsted base DBU, which was essential to achieve high stereoselectivity in the products.

**Scheme 14:**
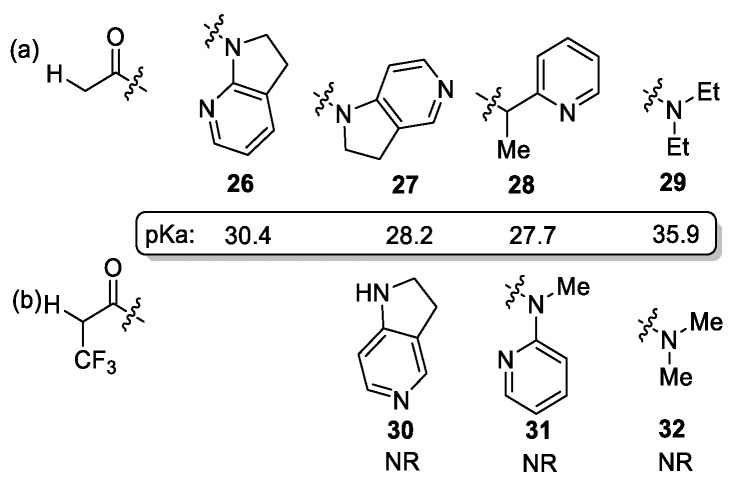
(**a**) The pKa values in DMSO for amides **26**–**29**; (**b**) the aldol reaction of ineffectiveness of α-CF_3_ amides with arylglyoxal hydrates.

To understand the key role of the 7-azaindoline group of amides in the success of this reaction, the authors also calculated the pKa values of a series of acetamides **26**–**29** with similar structure in DMSO using the density functional theory (DFT) method (Scheme 14a). The results showed that amides **26**–**28** have a higher acidity than their aliphatic counterparts **29**. Despite their having a much higher acidity than acetamide **26**–**29**, the *α*-CF_3_ amides **30**–**32** with different structures failed to produce the corresponding aldol product in the reaction using arylglyoxal hydrates as electrophiles (Scheme 14b). The results of these control experiments suggested that the 7-azaindoline moiety increased the acidity of the *α*-protons in the amides and facilitated enolization without causing undesired defluorination during the reaction.

**Scheme 15:**
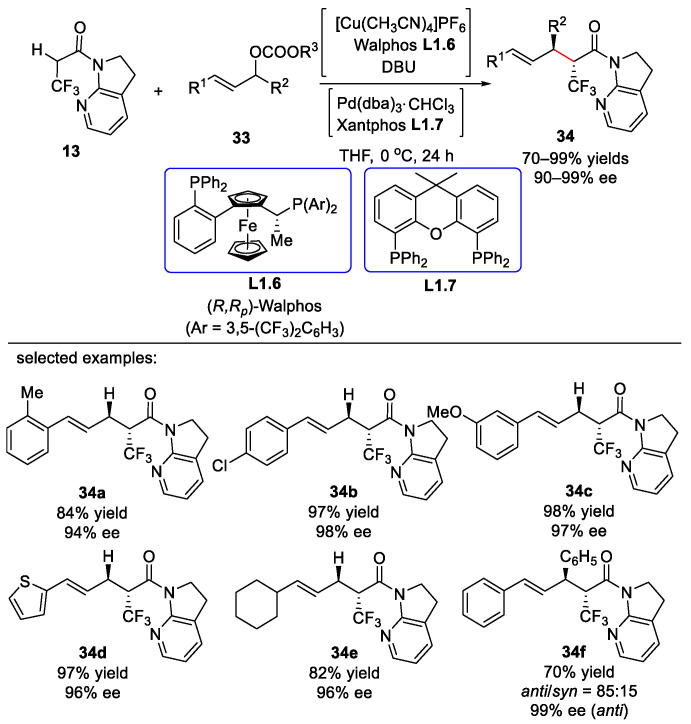
The Cu(I)/Pd(0) synergistic catalyzed *α*-allylation of an α-CF_3_ 7-azaindoline amide with allylic carbonates.

Continuing their efforts, in 2017, Shibasaki and co-workers proposed a strategy for the asymmetric *α*-allylation of *α*-CF_3_ 7-azaindoline amide **5** with allylic carbonates **33** under Cu/Pd synergistic catalysis (Scheme 15) [77]. In the Cu(I)/Pd(0) synergistic catalyst system, Cu(I)/DBU catalyzed the enolization of *α*-CF_3_ 7-azaindoline amide, while Pd(0) activated the allyl carbonate. In the Cu(I)/Pd(0) synergistic catalyst system, Cu(I)/DBU catalyzed the enolization of *α*-CF_3_ 7-azaindoline amide, while Pd(0) activated the allyl carbonate. The method showed good tolerance to the substituents of the substrate, and the reaction between allyl carbonates with various substituents and *α*-CF_3_ 7-azaindoline amide produced products **34** with at least one tertiary stereocenter in a high yield and with high enantioselectivity. It is worth noting that in the Cu(I)/Pd(0) synergistic catalyst system, 1,3-dissubstituted carbonate **33f** successfully reacted with *α*-CF_3_ 7-azaindoline amide to produce **34f** containing two successive tertiary stereocenters with good results (70% yield, 99% ee, and 85:15 dr).

**Scheme 16:**
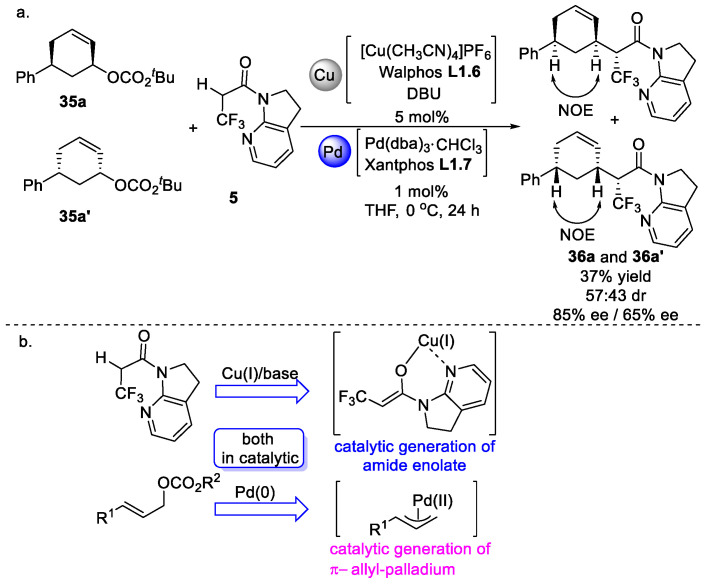
(**a**) The control experiment of α-CF_3_ 7-azaindoline amide with allylic carbonates; (**b**) the Cu(I)/Pd(0) catalyzed α-allylation of an α-CF_3_ 7-azaindoline amide.

A plausible pathway for the reaction of *α*-CF_3_ 7-azaindoline amide **5** and racemic carbonate **35** has been tentatively proposed, as shown in Scheme 16. They presumed that the Cu(I)/Pd(0) catalyst acted in a synergistic manner. The *α*-CF_3_ 7-azaindoline amide **5** reacted with diastereomers **35a** and **35a′** to provide products **36** and **36a′**, respectively, suggesting that a double inversion mechanism probably occurred in the *α*-allylation, in which the DBU in the Cu(I) catalytic system acted as a base to promote the removal of a hydrogen proton from the α-carbon atom of the *α*-CF_3_ 7-azaindoline amide **5**, and then the formed Cu(I) enolate acted as a soft nucleophile to attack the back of the π-allyl-Pd(II) intermediate. The stereochemical selectivity of the reaction is highly dependent on the chiral environment of the Cu(I) complex. The two catalysts Cu(I)/**L1.6** and Pd(0)/**L1.7** with phosphine ligands played the different roles in the *α*-allylation, as indicated by ^1^H, ^31^P, and ^19^F NMR analyses.

**Scheme 17:**
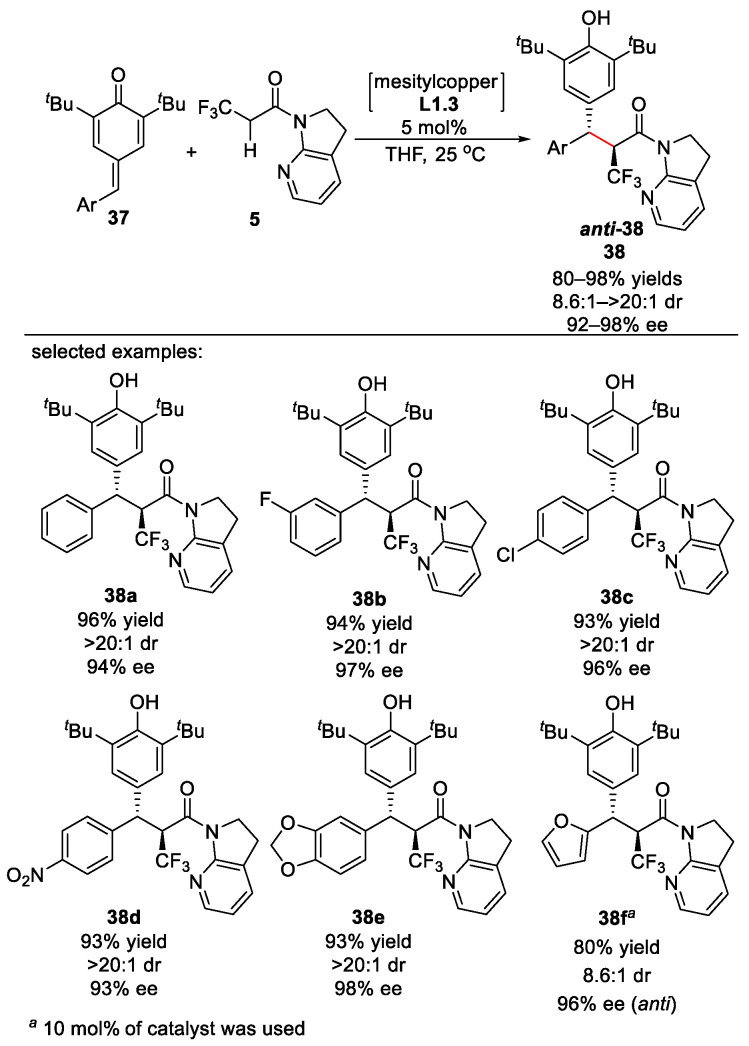
Asymmetric 1,6-conjugate addition of α-CF_3_ 7-azaindoline amide with p-QMs.

In their continuing program of the catalytic enolization chemistry of the *α*-CF_3_ 7-azaindoline amide, Shibasaki and colleagues developed the asymmetric 1,6-conjugate addition between *α*-CF_3_ 7-azaindoline amide **5** and *p*-quinone methides (*p*-QM) **37** catalyzed using mesitylcopper/**L1.3** as a cooperative catalyst (Scheme 17) [78]. The addition products **38**, containing two successive tertiary stereocenters, achieved good results (up to 98% yield, 20:1 dr, and 98% ee).

**Scheme 18:**
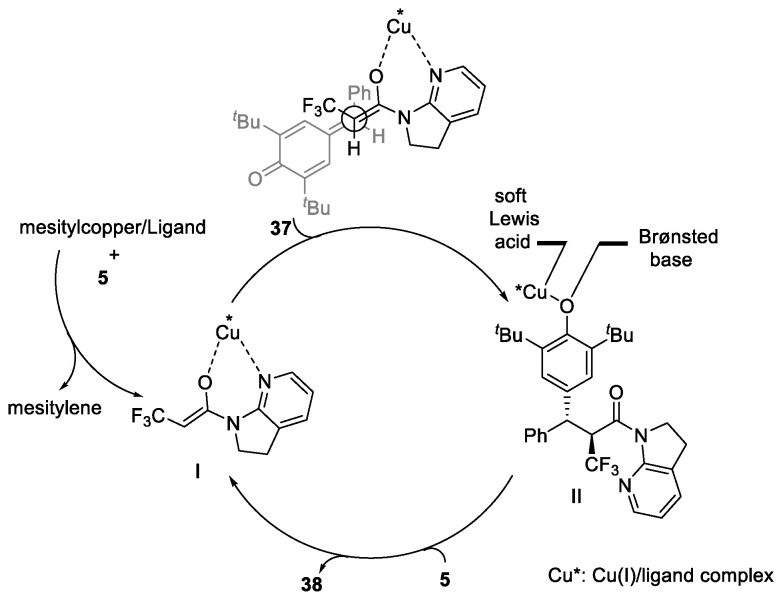
The plausible reaction pathway for the 1,6-conjugate addition of α-CF_3_ 7-azaindoline amide with *p*-QMs.

Moreover, a plausible pathway for the reaction of *α*-CF_3_ 7-azaindoline amide with *p*-QMs was proposed. As shown in Scheme 18, the mesitylcopper/**L1.3** is combined with *α*-CF_3_ 7-azaindoline amide **5** to form the Cu-enolate complex **I**, which is stabilized by binding interactions and decorated with the biaryl-type ligand. Then, the enolate oxygen anion of *α*-CF_3_ 7-azaindoline amide undergoes the 1,6-conjugate addition to *p*-quinone methides, affording the intermediate **II**, which acts as a soft Lewis acid/Brønsted base synergistic catalyst in the following catalytic cycle. Finally, the catalyst is released for the next cycle through the deprotonation of *α*-carbon of *α*-CF_3_ 7-azaindoline amide to provide the adduct **38**, in which the Cu(I)-aryl oxide moiety in the intermediate **II** served as the Brønsted base.

**Scheme 19:**
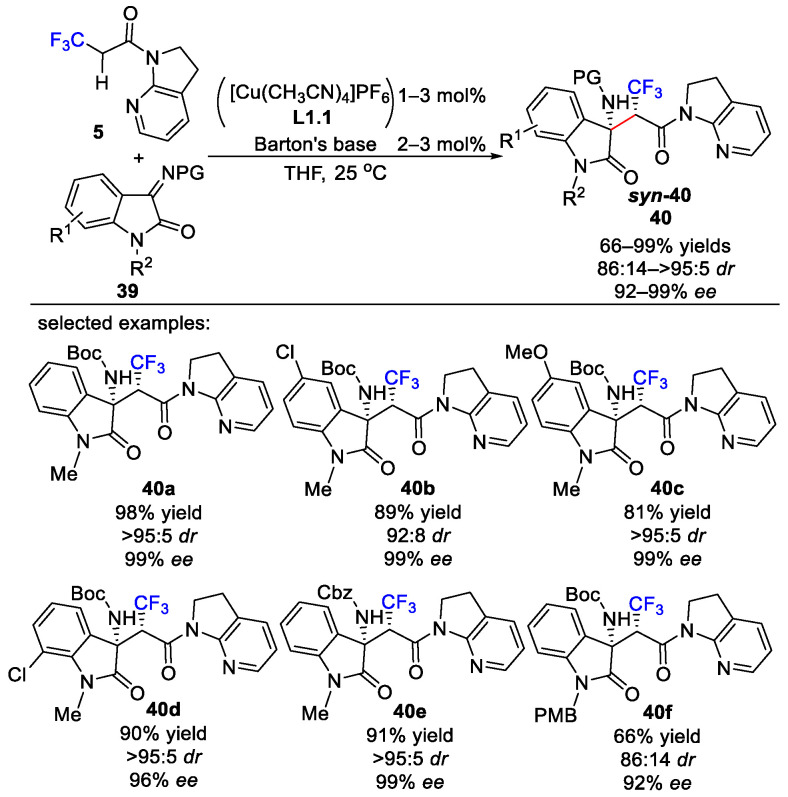
Asymmetric Mannich reaction of α-CF_3_ 7-azaindoline amide with isatin imines.

As part of their ongoing research on asymmetric reactions with *α*-CF_3_ 7-azaindoline amide **5** as the nucleophile under a soft Lewis acid/Brønsted base catalysis, Shibasaki’s group developed another asymmetric Mannich reaction of *α*-CF_3_ 7-azaindoline amide **5** with isatin imines **39** catalyzed by a Cu(I)/Ph-BPE ligand **L1.1**/Barton’s base complex, providing simple and convenient access to the Mannich adduct **40,** which contained two contiguous stereogenic centers, in 66–99 yields with 86:14→20:1 dr and 92–99% ee (Scheme 19) [79]. The substrate range was found to be quite broad. The potential application of this method was also demonstrated by the gram-scale reaction (1.46 g).

**Scheme 20:**
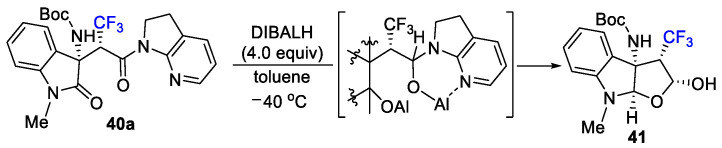
The transformation of **40a** into **41**.

To demonstrate the synthetic utility of the products, the conversions of Mannich adducts to other products are shown in Scheme 20. In the presence of DIBALH, the Mannich adduct **40a** was subjected to a triple-bond formation process to provide a multisubstituted tricyclic product **41** in 46% yield with 94:6 *dr*. This process included two reductions and one cyclization. Specifically, an aluminum alkoxide was formed via reduction of the oxindole moiety, the 7-azaindoline group was reduced to a masked aldehyde rather than an overreduced product, and the cyclization between aluminum alcohol and aldehyde resulted in product **41**.

### 2.3. α-Azido 7-Azaindoline Amide as a Nucleophile

Enantio-enriched *β*-hydroxy-*α*-amino acid derivatives, which are functionalized *α*-amino acids and are commonly found in both natural products and biologically active molecules, can be used to synthesize numerous important chiral compounds. The *β*-hydroxy-*α*-azido amide compounds can be regarded as *β*-hydroxy-*α*-amino acid derivatives.

**Scheme 21:**
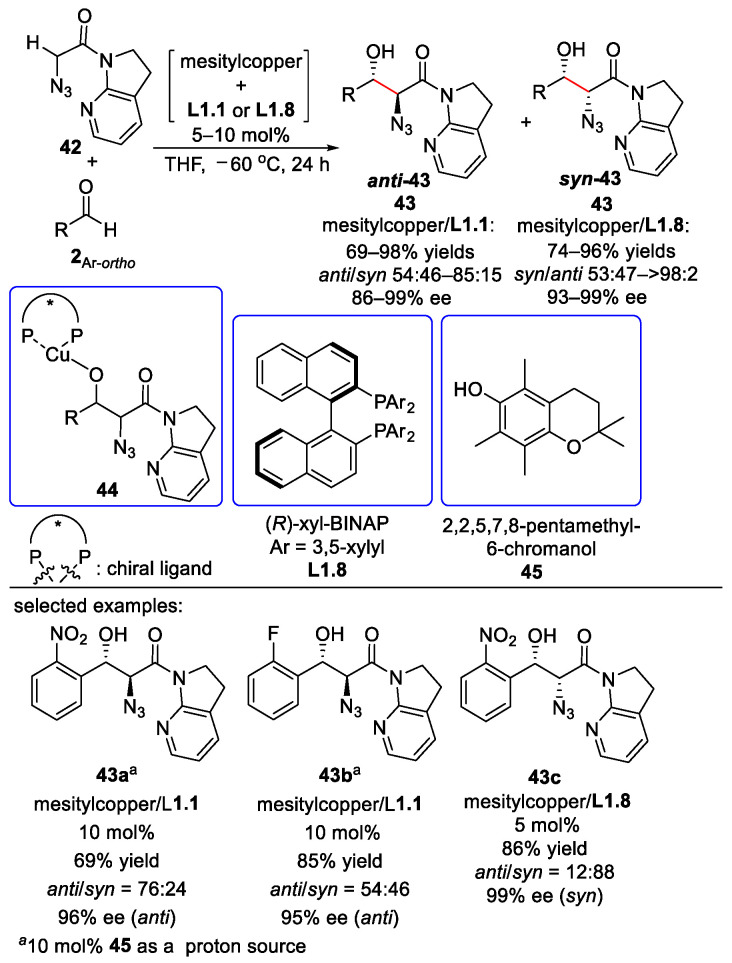
Asymmetric aldol reaction of an α-N_3_ 7-azaindoline amide with ortho-substituted aromatic aldehydes.

**Scheme 22:**
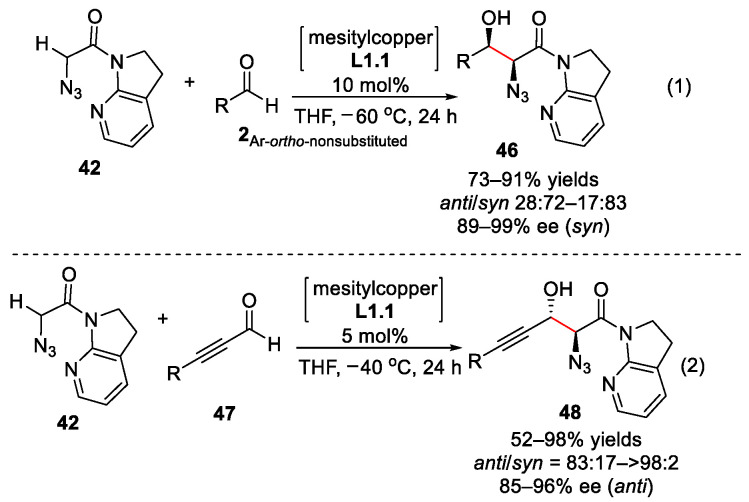
Asymmetric aldol reaction of an α-N_3_ 7-azaindoline amide with *ortho*-nonsubstituted aromatic aldehydes or ynals.

In 2015, Shibasaki’s group reported the asymmetric aldol reaction of *α*-azido 7-azaindoline amide **42** with aldehydes **2,** catalyzed by the mesitylcopper/ligand complex, which produced a series of *β*-hydroxy-*α*-amino acid derivatives bearing two adjacent tertiary stereocenters (Scheme 21) [80]. More specifically speaking, the *anti*-adducts **43** were obtained with 86–99% ee using mesitylcopper/**L1.1** catalyst, and the *syn*-adducts **43** were obtained with 93–99% ee using mesitylcopper/**L1.8** catalyst through the asymmetric aldol reaction of *α*-azido 7-azaindoline amide **42** with *ortho*-substituted aromatic aldehydes **2**. The authors also demonstrated that success in the mesitylcopper/**L1.1** catalytic asymmetric aldol reaction of *α*-azido 7-azaindoline amide **42** with *ortho*-nonsubstituted aromatic aldehydes **2** provided an effective method for the construction of *syn*-*β*-hydroxy-*α*-azido amides **46** (Scheme 22, Formula (1)). Unfortunately, the current catalytic systems were not applicable to other aldehydes, such as aliphatic aldehydes and *α*,*β*-unsaturated aldehydes, due to their lower reactivity. Notably, the *anti*-adducts **48** bearing a propargyl unit were also generated via the asymmetric aldol reaction between *α*-azido 7-azaindoline amide **42** and ynals **47** using mesitylcopper/**L1.1** catalyst (Scheme 22, Formula (2)).

From the experimental results, the following conclusions can be drawn: (i) The aldol reaction involving *ortho*-substituted aromatic aldehydes and ynals is carried out in a *trans*-selective manner using mesitylcopper/**L1.1** as the catalyst; (ii) The configuration of the stereocenter at the *α*-position of the adducts was switched via adjustments of the chiral ligand [**L1.1**: 2*S*, **L1.8**: 2*R*], regardless of the type of aldehyde used. In addition, both the reactions of *ortho*-nonsubstituted aromatic aldehydes catalyzed by mesitylcopper/**L1.8** and *ortho*-substituted aromatic aldehydes catalyzed by mesitylcopper/**L1.1** or mesitylcopper/**L1.8** prefer *syn*-adducts through the six-membered transition state. However, the reaction of *ortho*-substituted aromatic aldehydes catalyzed by mesitylcopper/**L1.1** forms *anti*-adducts **43** through an open transition state, likely due to the large steric effect.

**Scheme 23:**
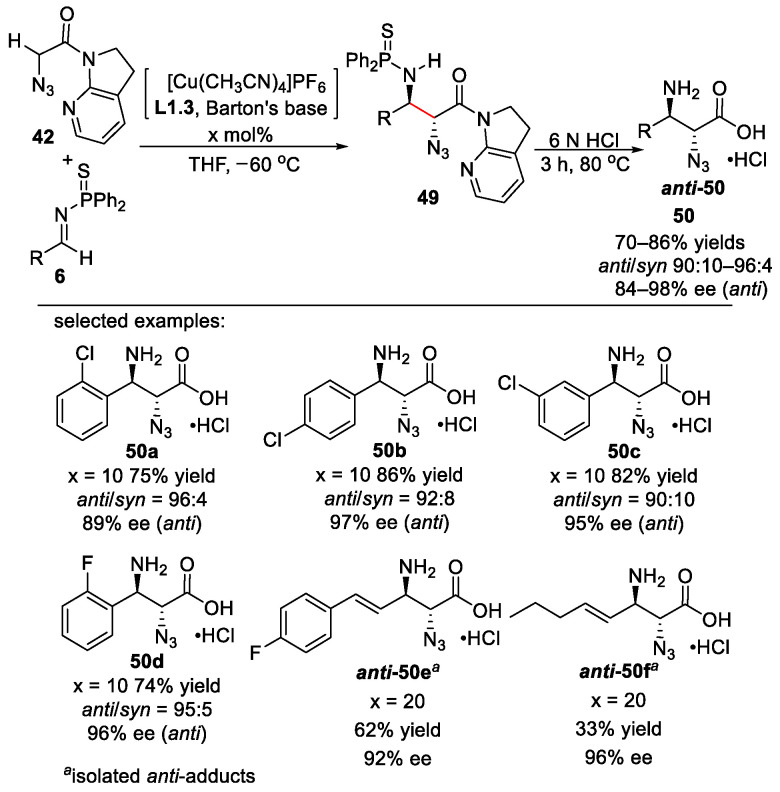
Asymmetric Mannich reaction of an α-N_3_ 7-azaindoline amide with imines.

To further explore the important role of *α*-azido 7-azaindoline amide in the synthesis of *α*-amino acid derivatives by direct enolization chemistry, Shibasaki’s group developed an asymmetric Mannich reaction of *α*-azido 7-azaindoline acetamide **42** and *N*-thiophosphinoyl imines **6** with Cu(CH_3_CN)_4_]PF_6_/(*R*)-xyl-Segphos **L1.3/**Barton’s base as a catalyst, affording Mannich adducts **49** containing both 7-azaindoline amide moiety and *N*-thiophosphinoyl amide fragment. Product **49** was easily hydrolyzed in acidic conditions, providing *anti*-*β*-amino-*α*-azido acid derivatives **50** with 84–98% ee (Scheme 23) [81]. This catalytic system was quite general for aromatic aldehydes with different substituents. The *o*-Cl-substituted imine gave the product **50a** with a slightly lower ee value (89%) than the other cases of Cl-substitution positions. Compared to the case of the *o*-Cl substituted imine, an improved ee value (96%) for the adducts **50d** was obtained by the reaction of the *o*-F-substituted imine as Mannich receptors, indicating that the decrease in the steric hindrance of imines positively influenced the enantioselectivity of the reaction. Moreover, other *anti*-*β*-amino-*α*-azido acids **50** derived from the reaction of *α*-azido 7-azaindoline acetamide and aliphatic imines could be obtained in 33–62% yields and 92–96% ee using increased catalyst loadings.

**Scheme 24:**
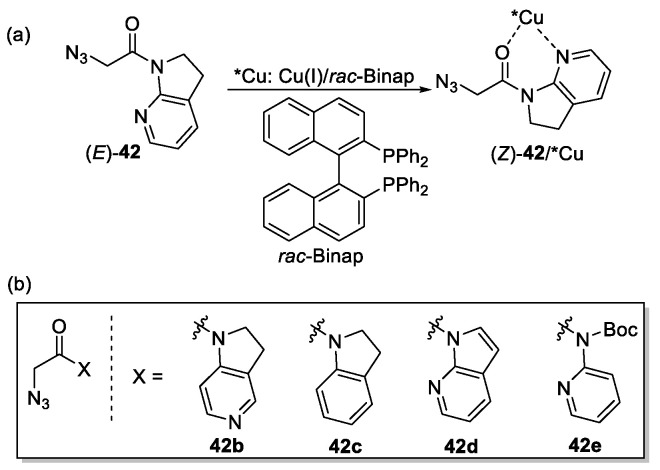
(**a**) (*E*)-α-N_3_ 7-azaindoline amide bidentate coordination to the Cu(I) complex; (**b**) structure of α-N_3_ amides with low activity.

The results of NMR spectroscopy (^1^H and ^15^N) showed that (*E*)-**42** was converted to (*Z*)-**42** after coordinating with Cu(I)/*rac*-Binap, and the azide group of *α*-azido 7-azaindoline amide did not coordinate with the Cu(I) complex (Scheme 24a). In addition, structurally similar *α*-azido amides **42b**–**e** failed to react with *N*-thiophosphinoyl imines **6** in the (Cu(CH_3_CN)_4_]PF_6_/**L1.3/**Barton’s base catalysis because the bidentate coordination of 7-azaindoline amide and Cu(I) was not possible in these cases. The results showed that the 7-azazindoline group in *α*-azido 7-azaindoline amide plays a key role in activating the substrate and controlling the stereoselectivity of the reaction (Scheme 24b).

**Scheme 25:**
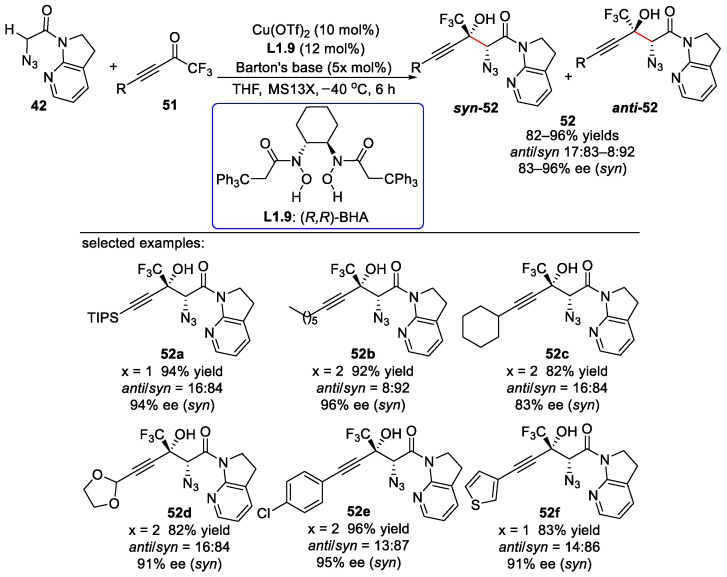
Asymmetric aldol reaction of an α-N_3_ 7-azaindoline amide with α-trifluoromethyl ynones.

**Scheme 26:**
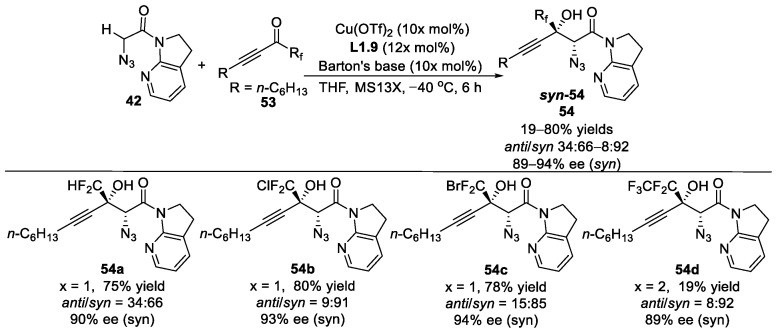
Asymmetric aldol reaction of the α-N_3_ 7-azaindoline amide with other *α*-fluorinated ketones.

To further study the utility values of *α*-N_3_ 7-azaindoline amide in the synthesis of a crucial class of chiral building blocks, Shibasaki and co-workers subsequently explored the asymmetric aldol reaction between *α*-N_3_ 7-azaindoline amide **42** and *α*-trifluoromethyl ynones **51** using Cu(OTf)_2_/(*R*,*R*)-BHA**L1.9**/Barton’s base as catalysts and MS13X as an additive, providing trifluoromethyl substituted propargylic tertiary alcohols **52** in 82–96% yields and 17:83–8:92 dr and 83–96% ee (Scheme 25) [82]. Triisopropylsilyl (TIPS)-substituted trifluoromethyl ynones reacted smoothly with *α*-N_3_ 7-azaindoline amide **42** to provide *syn*-**52a** in 94% ee. The substrates of trifluoromethyl ketones with alkyl (*cyclo*-C_6_H_13_) on the alkynyl group were tolerated. However, a slightly lower ee value (**52b** vs. **52c**) was observed in the case of *cyclo*-C_6_H_13_ ynones than in the case of *n*-C_6_H_13_ ynones. It was also found that other *α*-fluorinated ketones were appropriate for this aldol reaction with high efficiency. The substrate **53d**, which carries a CF_2_CF_3_ group in the *α*-position of ynone, reacted with *α*-N_3_ 7-azaindoline amide to provide the adduct **54d** in 89% ee, but in low yield (only 19%), even when the catalyst loading was increased to 20 mol% (Scheme 26).

**Scheme 27:**
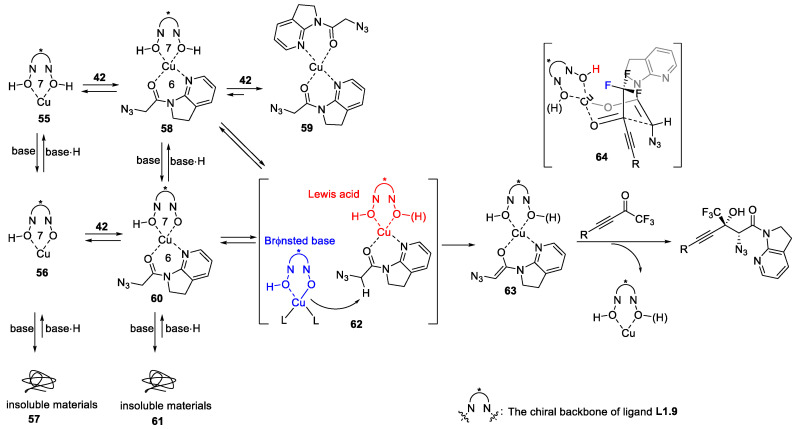
A proposed pathway for the aldol reaction of α-N_3_ 7-azaindoline amide and α-trifluoromethyl ynones.

According to the results of the control experiment, the mechanism of the asymmetric aldol reaction between an *α*-N_3_ 7-azaindoline amide and *α*-trifluoromethyl ynones was explored (Scheme 27). In the current catalytic system, consisting of Cu(OTf)_2_/(*R*,*R*)-BHA **L1.9**/Barton’s base, equivalent amounts of Cu(OTf)_2_ and the ligand **L1.9** form a 7-membered chelate complex **55**. When the *α*-N_3_ 7-azaindoline amide was added to the complex **55** solution, a precipitate complex **59** with a 1:2 Cu/amide but no ligand was formed. The Barton’s base is usually used for the deprotonation and enolization of *α*-N_3_ 7-azaindoline amide, but the excessive amount of Barton’s base led to the deprotonation of the ligand **L1.9**. Thus, the undesired species (**56** and **57**) were also formed in the presence of excess Barton’s base in the solution of complex **55**. In particular, the formation of **57** is irreversible. An unstable complex **58** and a slightly cloudy solution **60** were also obtained when the *α*-N_3_ 7-azaindoline amide **42** was added to the solution of complex **55** and **56**, respectively. The hydroxamic acids (**L1.9**) deprotonated in the presence of Barton’s base and became more strongly bound ligands. The solution **60** was converted into insoluble substances **61** using another equivalent of Barton’s base. Therefore, a higher loading of Barton’s base was not good for the catalytic system because the insoluble materials (**57**, **61**) were formed. The additive MS13X has a suitable pore structure to reserve *α*-N_3_ 7-azaindoline amide and Barton’s base, so that the pathways used to form the **57**, **59**, and **61** were suppressed. The authors presumed that the Cu(II)/L1.9 complex should play a bifunctional role. As shown in **62**, the Cu(II) moiety in the complex served as a Lewis acid to activate *α*-N_3_ 7-azaindoline amide **42** via intermolecular bonding, affording the compound of Cu(II)/**L1.9**/amide. Simultaneously, the proton-deficient moiety of Cu(II)/**L1.9** served as a Brønsted base to remove the *α*-proton of *α*-N_3_ 7-azaindoline amide in Cu(II)/**L1.9**/amide. In other words, the proton-deficient Cu(II)/**L1.9** complex promoted the enolization of *α*-N_3_ 7-azaindoline amide **42**. The high stereoselectivity of the aldol reaction likely benefited from non-bonding interactions (such as hydrogen bonding or ion–dipole interactions) between the fluorine atoms in the ynones **51** and the acidic protons in the ligand, as illustrated in complex **64**.

**Scheme 28:**
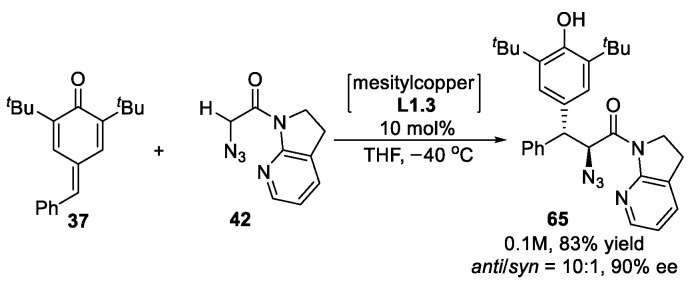
Asymmetric 1,6-conjugate addition of *α*-N_3_ 7-azaindoline amide with *p*-QMs.

In 2018, Shibasaki and co-workers developed an highly effective asymmetric 1,6-conjugate addition of *α*-N_3_ 7-azaindoline amide **42** to *p*-quinone methides (*p*-QMs) **37** [78]. As shown in Scheme 28, the adduct **65** was synthesized in 83% yield, 10:1 (*anti*/*syn*) dr and 90% ee using the mesitylcopper/chiral ligand **L1.3** complex as a catalyst at −40 °C. This is a part of the research on the asymmetric 1,6-conjugate addition of *α*-substituted amides (CF_3_, N_3_, Me, and OBn) to *p*-QMs.

### 2.4. α-Alkyl Substituted 7-Azaindoline Amide as a Nucleophile

**Scheme 29:**
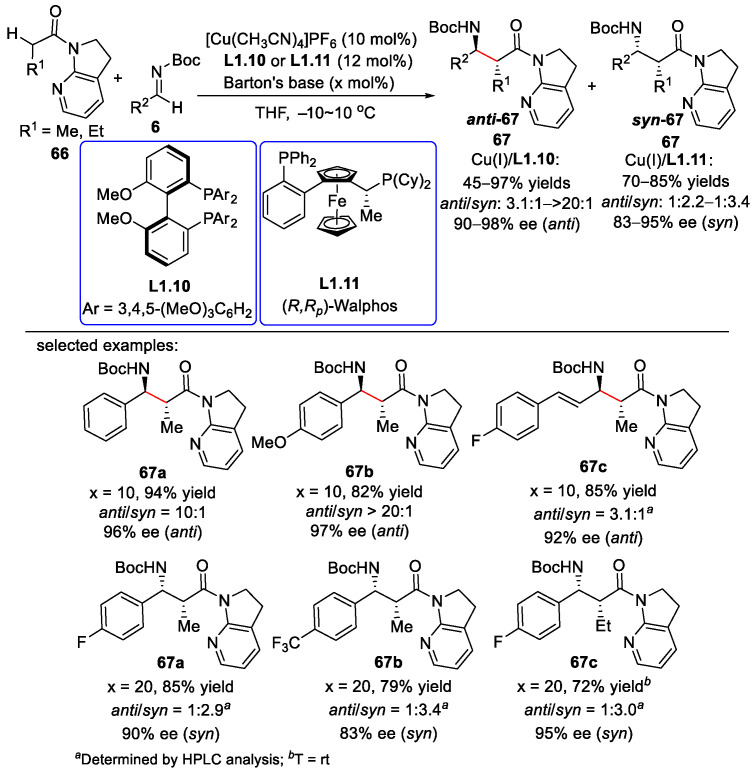
Asymmetric Mannich reaction of *α*-alkyl 7-azaindoline amides and imines.

In 2016, Shibasaki’s group reported the direct asymmetric Mannich reaction of *α*-alkyl 7-azaindoline amide **66** with *N*-Boc imines **6** using a Lewis acid/Brønsted base cooperative catalyst (Scheme 29) [83]. Interestingly, two different configurations of *β*-amino acid derivatives (*anti*-**67** and *syn*-**67**) were obtained with good results via modulation of the type of ligand. Specifically, the catalyst consisting of [Cu(CH_3_CN)_4_]PF_6_/**L1.10**/Barton’s base was applied to the Mannich reaction between *α*-alkyl 7-azaindoline amide and imines at −10 °C, providing *anti*-**67** with two tertiary stereocenters with 90–98% ee. Adduct **67** was converted into an α-methyl-β-amino acid via treatment with 6 M HCl in MeOH at 80 °C. They also developed the [Cu(CH_3_CN)_4_]PF_6_/**L1.11**/Barton’s base for the asymmetric synthesis of *β*-amino acid derivatives through the reaction between amide and electron-deficient imines, providing the *syn*-**67** with 83–95% ee.

**Scheme 30:**
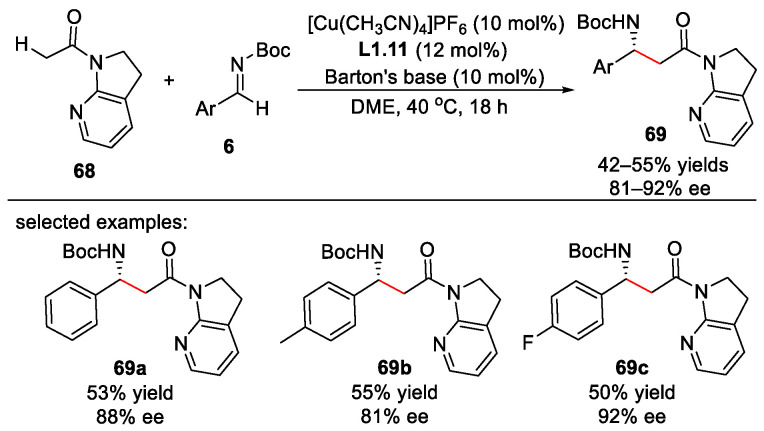
Asymmetric Mannich reaction of the 7-azaindoline acetamide with imines.

Shibasaki and coworkers further extended this catalytic system to 7-azaindoline acetamide **68** and found that the [Cu(CH_3_CN)_4_]PF_6_/**L1.11** complex was effective with DME as a solvent. The reaction between 7-azaindoline acetamide **68** and 1.5 equivalents of *N*-Boc imine **6** proceeded smoothly, affording the Mannich adducts **69** bearing a chiral *β*-aminoacetate unit with 81–92% ee but only 42–55% yields (Scheme 30) [83]. This extended catalytic system was helpful to develop the application of acetamides via enolization in the asymmetric Mannich reaction, because products **69** tend to participate in enolization.

**Scheme 31:**
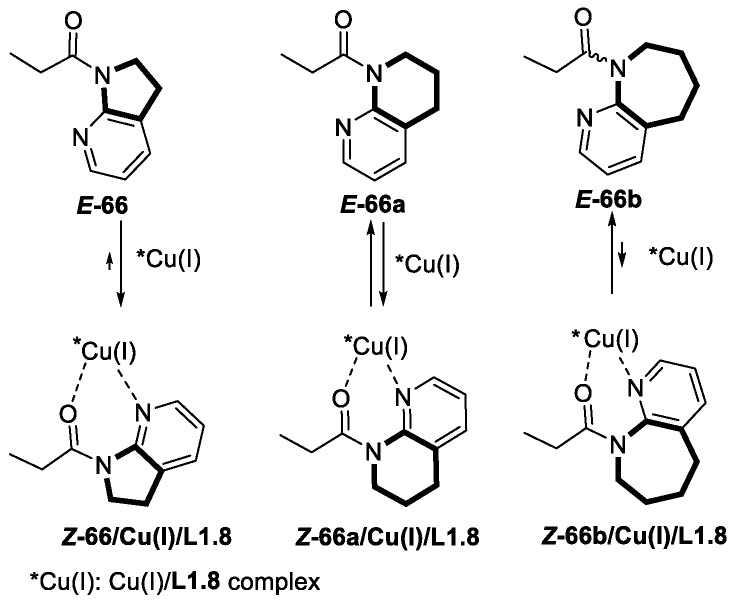
Coordination of amides with the Cu(I)/**L1.8** complex.

The authors also investigated the coordination of propionamides with the Cu(I)/**L1.8** through NMR research, which showed that 7-azaindoline propionamide binds to the Cu(I)/**L1.8** via bidentate coordination, with the conformation changing from *E* to *Z*. In contrast, amides **66a**-**b**, structurally similar to 7-azaindoline amide **66**, failed to form a bidentate coordination with the Cu(I) complex. The NMR results revealed that 50% of **66a** formed the *Z*-**66a**/Cu(I)/**L1.8** complex, whereas **66b** scarcely afforded the corresponding *Z*-**66b**/Cu(I)/**L1.8** complex. The difference in reactivity mainly depended on the coordination capacity of the amide with the Cu(I)/**L1.8** complex (Scheme 31).

**Scheme 32:**
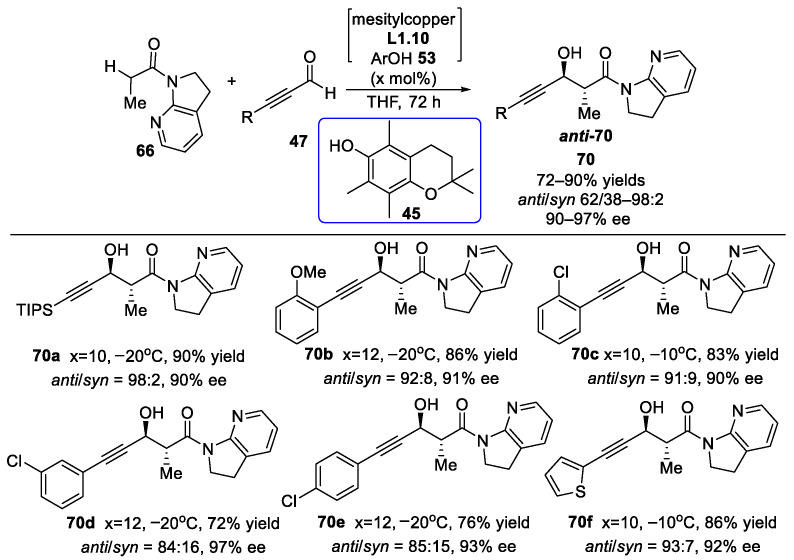
Asymmetric aldol reaction of propionamide with ynals.

**Scheme 33:**
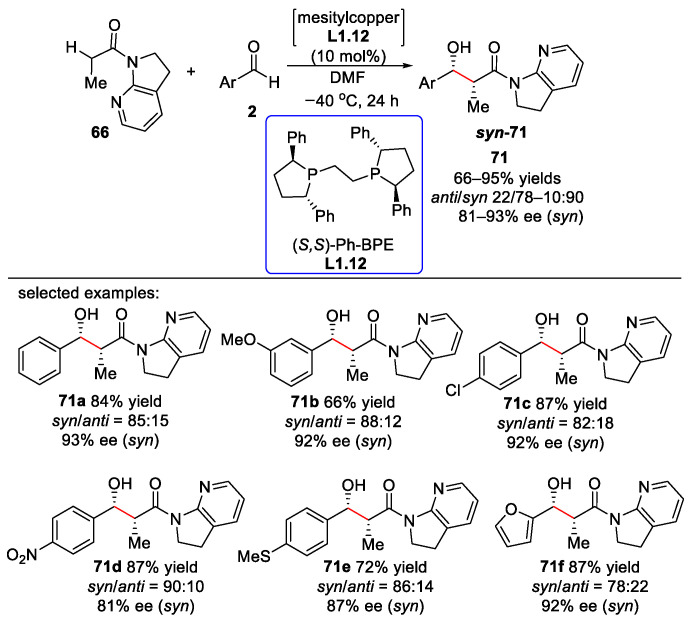
Asymmetric aldol reaction of propionamide with aromatic aldehydes.

To further expand the scope of *α*-alkyl 7-azaindoline propionamide in direct catalytic asymmetric addition reactions, Shibasaki and co-workers explored the performance of *α*-alkyl 7-azaindoline propionamide **66** in the asymmetric aldol reaction [84]. The reaction in which the ynals **47** and aromatic aldehydes **2** were used as electrophiles proceeded smoothly and provided the aldol adducts with good results. The reaction, in which the ynals **47** and aromatic aldehydes **2** were used as electrophiles, proceeded smoothly and provided the aldol adducts with good results. As shown in Scheme 32, the reaction of *α*-methyl 7-azaindoline amide **66** and ynals **47** was catalyzed by mesitylcopper/**L1.10** with ArOH **45** as the proton source, affording the *anti*-**70** containing the propargylic alcohol unit with 90–97% ee. The desired adducts **70** were efficiently obtained regardless of the electronic properties and the positions of the substituents on the phenyl ring, probably because the phenyl of the ynals was far away from the reaction site. The aromatic aldehydes **2** were also employed as nucleophiles, providing a series of *β*-hydroxy propionate derivatives *syn*-**71** with 81–93% ee under the catalysis of the mesitylcopper/**L1.12** complex (Scheme 33).

**Scheme 34:**
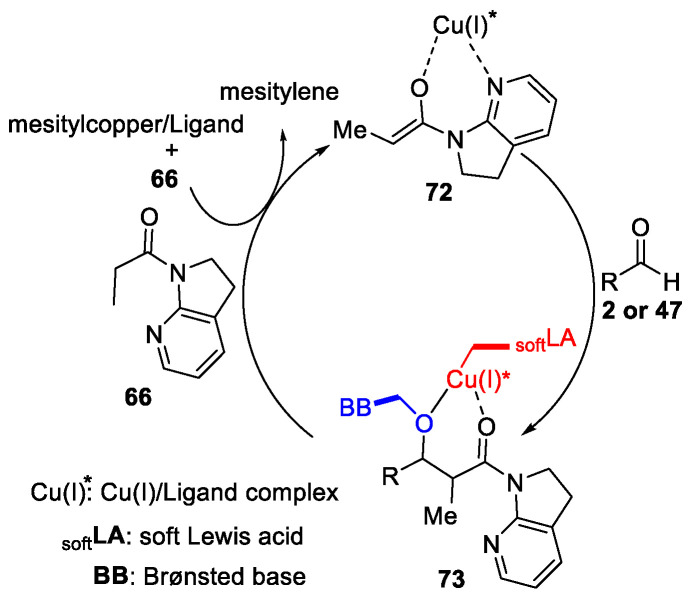
Proposed reaction pathway for Cu(I)-catalyzed reaction of *α*-methyl 7-azaindoline amide and aldehydes.

A plausible catalytic cycle process for the reaction of *α*-methyl 7-azaindoline amide and aldehydes was put forward, as shown in Scheme 34. Firstly, *E*-configuration *α*-methyl 7-azaindoline amide **66** was combined with the mesitylcopper/ligand to form *Z*-configuration amide/Cu(I)/enolate **72** via bidentate coordination, which controlled the chiral environment in the aldol reaction. Then, an aldol addition occurred between the aldehydes (**2** or **47**) and complex **72** to form Cu(I) aldolate **73**. Finally, the Cu(I) aldolate **73**, acting as a Brønsted base, deprotonated *α*-methyl 7-azaindoline amide **66** to provide the desired product.

**Scheme 35:**
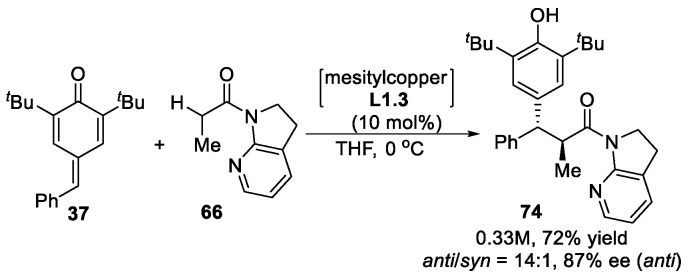
Asymmetric 1,6-conjugate addition of *α*-Me 7-azaindoline amide with *p*-QMs.

In the work by Shibasaki’s research group in 2018, a series of *α*-substituted 7-azaindoline amides (CF_3_, N_3_, Me, and OBn) were employed as donors to react with *p*-QMs **37** for the asymmetric synthesis of the desired adducts bearing a diarylmethane unit [78]. The *α*-methyl 7-azaindoline amide **66** also participated in the 1,6-conjugate addition catalyzed by the mesitylcopper/**L1.3** complex, affording the product **74** with 87% ee (Scheme 35).

**Scheme 36:**
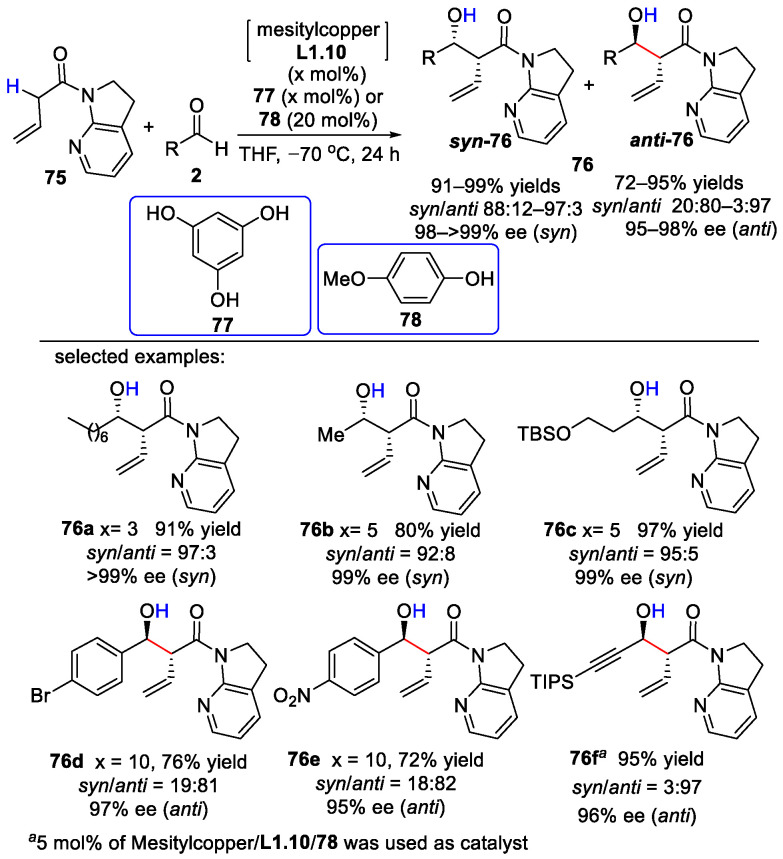
Asymmetric aldol reaction of *α*-vinyl 7-azaindoline amide with aliphatic and aromatic aldehydes.

Motivated by the successful application of the mesitylcopper/**L1.10** complex as a catalyst in the asymmetric addition of *α*-methyl 7-azaindoline amide **66** to aromatic aldehydes **2** and ynals **47** (Scheme 32 and Scheme 33), Shibasaki et al. applied the same complex to the asymmetric aldol reaction of *α*-vinyl 7-azaindoline acetamide **75** with both aliphatic and aromatic aldehydes [85]. When phloroglucinol **77** was used as an additive, *α*-vinyl 7-azaindoline acetamide **75** as a nucleophile reacted with aliphatic aldehydes **2** to give adducts *syn*-**76** bearing contiguous tertiary stereocenters with 98→99% ee (Scheme 36). The mesitylcopper/**L1.10** complex was also suitable for aromatic aldehydes by switching the additive **77** to **78**, affording the desired products *anti*-**76** with 95–98% ee (Scheme 36). From the above results, we reached the conclusion that the *α*-vinyl 7-azaindoline acetamide **75** reacted with aromatic aldehydes in an *anti*-selective manner using (*R*)-trimethoxy-Biphep **L1.10** as a chiral ligand, while, complementarily, the *α*-Me 7-azaindoline acetamide reacted with aromatic aldehydes in a *syn*-selective manner by employing (*S*,*S*)-Ph-BPE **L1.12** as a chiral ligand. The catalytic system also proved to be almost unbiased for the ynal, affording adduct **76f** with good results.

**Scheme 37:**
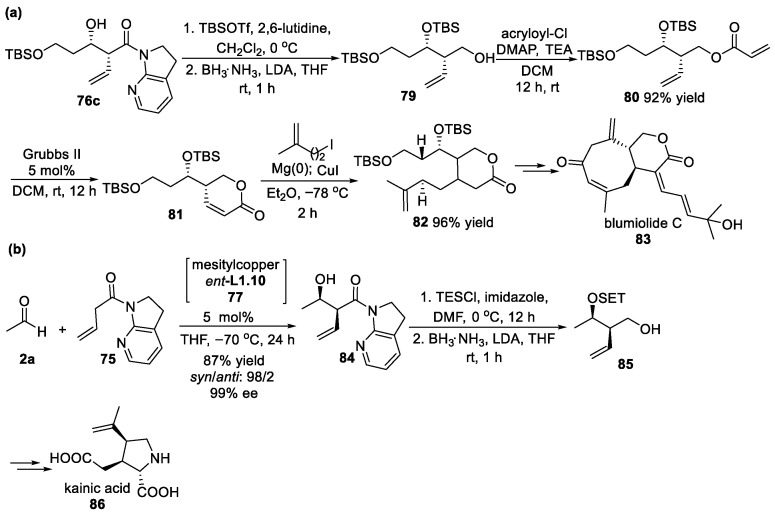
(**a**) An adduct **76c** used in the synthesis of the key intermediate **82** of the Blumiolide C **83**; (**b**) an adduct **84** used in the synthesis of the key intermediate **85** of kainic acid **86**.

The 7-azaindoline moiety of adducts **76** was easily converted, which helped to synthesize the key intermediate of blumiolide C **83** and kainic acid **86**. Treatment of the adduct **76c** with TBSOTf, followed by Myers’ reduction of the 7-azaindoline group of **76c**, afforded the primary alcohol **79** with 97% yield. The unsaturated valerolactone **80** was smoothly obtained by esterification with acryloyl chloride followed by ring-closing metathesis with the second-generation Grubbs catalyst. The key intermediate **82** for blumiolide C **83** was received with 96% yield via the conjugate addition of **81** and 4-iodo-2-methylbut-1-ene (Scheme 37a). The reaction of acetaldehyde **2a** with *α*-vinyl 7-azazoline acetamide **75** was catalyzed by mesitylcopper/*ent*-**L1.10**/phloroglucinol, resulting in the formation of *syn*-**84**, which is the enantiomer of **76b**. The adduct **84** was silicified and reduced to produce intermediate **85**, which was essential for the final synthesis of the kainic acid **86** (Scheme 37b). The importance lies in that the kainic acid **86** is a type of natural marine product and has biological applications because of the biological activities such as neuroexcitatory, insecticidal, and anthelmintic properties.

**Scheme 38:**
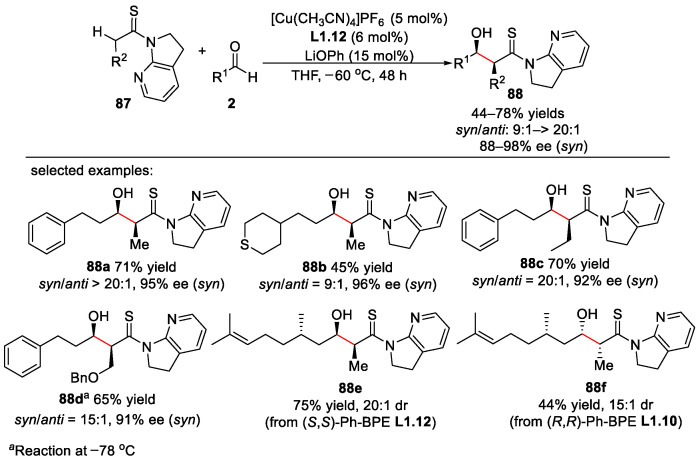
Asymmetric aldol reaction of 7-azaindolinyl thioamide with aldehydes.

In their continuing study of catalytic enolization chemistry, the direct catalytic asymmetric aldol reaction of 7-azaindolinyl thioamides **87** with aliphatic aldehydes **2** in the presence of the [Cu(CH_3_CN)_4_]PF_6_/(*S*,*S*)-Ph-BPE **L1.12**/LiOPh was disclosed by Shibasaki and co-workers in 2020 (Scheme 38) [86]. In this reaction, a variety of aldol products **88** bearing two consecutive stereocenters were successfully obtained with moderate to good yields (44–78%), with 9:1→20:1 dr and 88–98% ee (*syn*). In this catalytic system, the scope of aldehydes as substrates has been greatly expanded. When 7-azaindolinyl thioamides **87** were reacted with 3-phenylpropanal, no self-aldol product was formed and *syn*-**88a** was obtained with 95% ee. Notably, (-)-citronellal reacted with thioamides **87** to provide the target products **88e**-**f** with a similar diastereoselectivity, regardless of whether (*S*,*S*)-Ph-BPE **L1.12** or (*R*,*R*)-Ph-BPE **L1.1** was used, indicating that the chiral environment of the *Z*-enolate determined the diastereoselectivity of the aldol reaction.

**Scheme 39:**
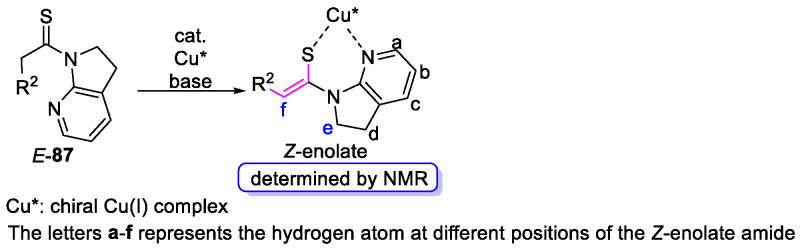
Formation of *Z*-enolate determined by NMR.

According to the NMR spectroscopy results, the proton (H*_f_*) of the enolate and the protons (H*_e_*) of the pyrroline moiety were identified by remarkable NOE signals, suggesting that the reaction of mesitylcopper with (*E*)-*α*-alkyl 7-azaindolinyl thioamide **87** provided the corresponding *Z*-configured thioamide enolate. The 7-azaindoline group had sufficient capacity to prevent rotation from the C(py)-N(amide) bond and stabilize the thioamide enolate via Cu(I)/ligand coordination (Scheme 39).

### 2.5. α-Halo Substituted 7-Azaindoline Amide as Nucleophiles

**Scheme 40:**
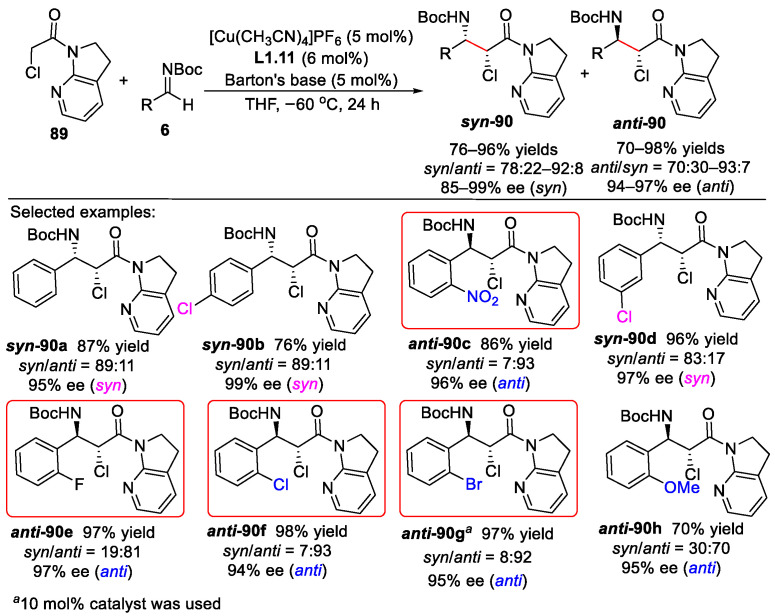
Cu(I)-catalyzed asymmetric Mannich of *α*-Cl 7-azaindoline amide **89** with monosubstituted N-Boc imines.

**Scheme 41:**
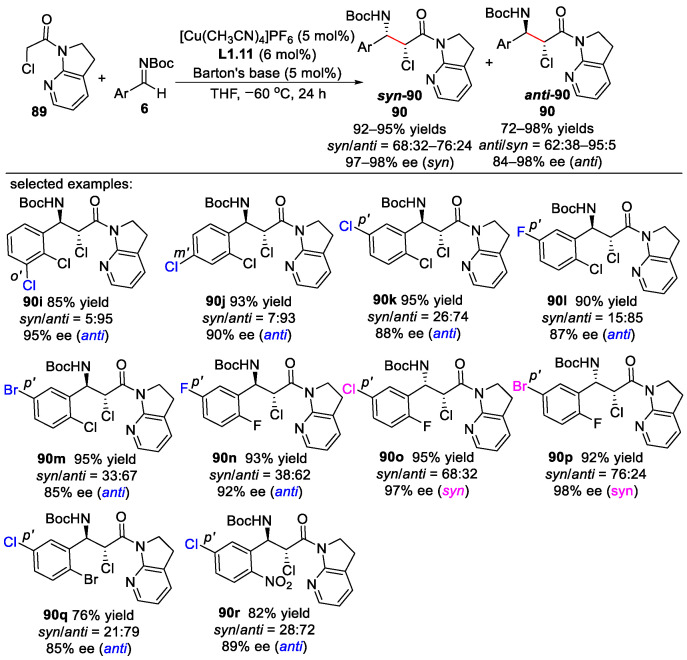
Cu(I)-catalyzed asymmetric Mannich of *α*-Cl 7-azaindoline amide with disubstituted N-Boc imines.

In view of the wide application of the enolate chemistry of *α*-substituted 7-azaindoline amides, Shibasaki et al. were encouraged to investigate the reaction of *α*-halo (*α*-F, -Cl, -Br, -I) 7-azaindoline amides as potential enolates with imines. They achieved a direct catalytic asymmetric Mannich reaction between *α*-Cl 7-azaindoline amide **89** and *N*-carbamoyl imines **6** without undesirable dehalogenation, using [Cu(CH_3_CN)_4_]PF_6_/(S,S)-Ph-BPE **L1.11**/Barton’s base as a synergistic catalyst (Scheme 40 and Scheme 41) [87]. The use of 5 mol% loading of the catalyst smoothly promoted the Mannich reaction at −60 °C, affording adducts containing halogen atoms on the stereogenic carbon in high yields with moderate to good stereoselectivities. In their unremitting efforts, the authors extended the range of *α*-Cl substituted adducts **90** via Mannich addition with monosubstituted *N*-Boc aromatic imines under the synergistic catalysis. Specifically, the *syn*-**90** were obtained with 85–99% ee by using a variety of monosubstituted imines without *o*-substituents, whereas the *anti*-**90** was received with 94–97% ee when the *o*-monosubstituted imines were reacted with the *α*-Cl 7-azaindoline amide **89**. In the series of adducts **90**, the *α*-chiral carbons of the *syn*-**90** and the *anti*-**90** were consistent in *S*-configuration, whereas the *β*-chiral carbons were inconsistent in configuration, indicating that the presence of imine *ortho*-substituents altered the selectivity of the prochiral face.

The authors further investigated the substituent effect to obtain an in-depth understanding of the reason for the change in diastereoselectivity. They explored the asymmetric Mannich reaction of *α*-Cl 7-azaindoline amide **89** and disubstituted *N*-Boc imines **6** catalyzed by the [Cu(CH_3_CN)_4_]PF_6_/(*S*,*S*)-Ph-BPE **L1.11**/Barton’s base. The adducts were obtained in 72–97% yields with 5:95–76:24 (*syn*/*anti*) dr and 84–98% ee using a variety of disubstituted imines with at least one *o*-substituent (Scheme 41). Compared to the *anti*-**90f**, the *m′*-Cl or *o′*-Cl substituted dichloro adducts *anti*-**90i**–**j** were obtained with a comparable stereoselectivity, but *p′*-Cl substituted dichloro adduct **90k** had a significantly reduced *anti*-selectivity. The *anti*-selectivity of the disubstituted imines was significantly influenced by the steric factor of the *p′*-substituents, which was suggested by the variation in selectivity from 15:85 (*syn*/*anti*) in the case of the *p′*-F substituted imine (**6l**) to 33:67 in the case of the *p′*-Br substituted imine (**6m**). In comparison, the *o*-F substituent (**6e**) had a lesser influence on the *anti*-diastereoselectivity (*syn*/*anti* = 19/81) compared to the *o*-Cl case (**6f**) (*syn*/*anti* = 7/93). Interestingly, the preferential *syn*-selectivity was more dependent on the anchoring effect in the cases of the *p′*-Cl substituted imine (**6o**) and *p′*-Br substituted imine (**6p**) than in the case of the *o*-F substituent. The preferential *syn*-selectivity of the *p′*-substituent was similar for the imines **6q-r** with *o*-Br-*p′*-Cl and *o*-NO_2_-*p′*-Cl, respectively (*anti*-**90g** vs. *anti*-**90q**, *anti*-**90c** vs. *anti*-**90r**). *Ortho*-substituted imines tend to synthesize *syn*-**90**, whereas non-*ortho*-substituted imines easily produce *anti*-**90**, as the presence of *ortho*-substituents alters the face selection of aromatic imines.

**Scheme 42:**
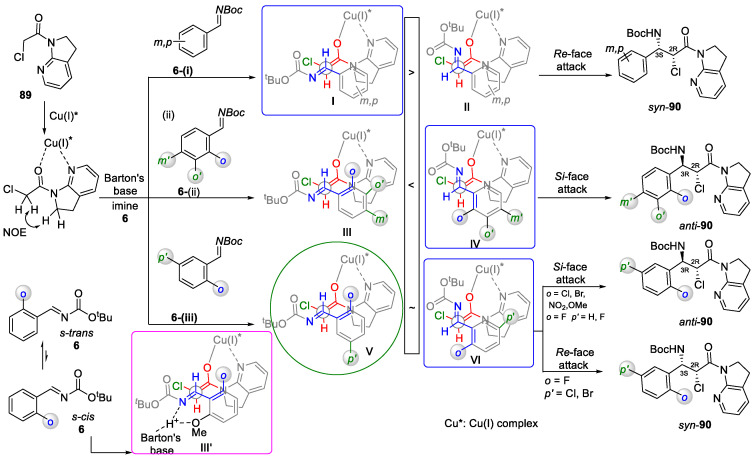
A probable rationale for the divergent diastereoselectivity of the Mannich reaction through various transition-state models shown in the squares.

Based on the experimental results, the authors proposed a reasonable explanation for the divergent diastereoselectivity (Scheme 42). Consistent with previous studies of various *α*-substituted 7-azaindoline amides, the *α*-Cl 7-azaindoline amide **89** exhibited an *E*-configuration in solution, and the configuration of **89** changed from an *E*-configuration to *Z*-configuration when the **89**/Cu(I) complex was afforded via **89,** which was coordinated with the Cu(I) complex. The corresponding enolate was obtained via deprotonation of the **89**/Cu(I) complex with Barton’s base, and subsequently underwent the asymmetric Mannich reaction with imine **6**. In case (i), under the catalysis of the Cu(I) complex, *α*-Cl 7-azaindoline amide **89** preferentially attacked the *Re*-face of *m*- or *p*-substituted imines **6**-(i) via a transition-state model **I** with the minimum steric hindrance and dipole moment, affording products **90b** and **90d** in a *syn*-selective manner. In the case (ii), imine **6**-(ii) tended to form an *s*-*trans* configuration rather than an *s*-*cis* configuration, due to the repulsion between the nitrogen atom and the *ortho* substituent of the imine. The *Si*-face of imine in *s*-*trans* configuration was more likely to be attacked to form a C–C bond via the transition-state model **IV**, providing the *anti*-configuration adducts *anti*-**90i**–**j**. The low *anti*-selectivity of the products **90h** was obtained via the reaction of the *o*-Ome-substituted imine with *α*-Cl 7-azaindoline amide **89**. This was attributed to the fact that both the nitrogen atom and the *o*-OMe group of the imine formed hydrogen bonds with the proton, which facilitated the attack on the *Re* face of the imine with *s*-*cis* configuration, thus providing the major product with *syn*-configuration via the transition-state model **III′**. In the case (iii), the major isomer with an *anti*-configuration was obtained by attacking the *Si*-face of the *p′*-substituted *o*-Cl imines **6k**–**m**, owing to the small repulsion of the *p′*-substituent steric hindrance via the transition-state model **VI**. Imines with larger substituents at the *p′* position, such as *o*-F-*p′*-Cl- or *o*-F-*p′*-Br-substituted imines **6o**–**p**, were conducive to form *syn*-**90o**–**p** via the transition-state model **V**, whereas the *o*-F*-p′*-F-substituted imine **6n** was conducive to forming *anti*-product **90n** via the transition-state model **IV**. In the reaction between the *o*-substituted *p′*-Cl imines **6q**–**r** and *α*-Cl 7-azaindoline amide **89**, the *anti*-products **6q**–**r** were more easily formed by attacking the *Si*-face of imines via the transition-state model **VI**.

**Scheme 43:**
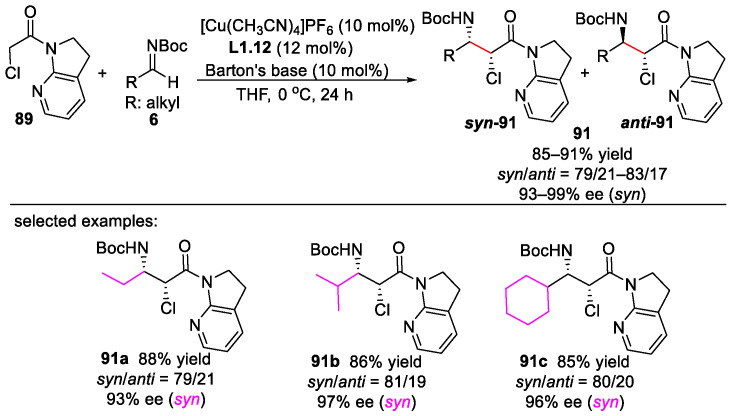
Cu(I)-catalyzed asymmetric Mannich of *α*-Cl 7-azaindoline amide **89** with aliphatic imines.

Aliphatic imines were also used as electrophiles to the asymmetric Mannich reaction with *α*-Cl 7-azaindoline amide **89**, providing the Mannich products **91** with 85–90% yield, 79:21–83:17 (*syn*/*anti*) dr, and 85–90% ee (*syn*), by switching the chiral ligand from **L1.11** to **L1.12** of the current Cu(I)/Barton’s base catalytic system (Scheme 43).

**Scheme 44:**
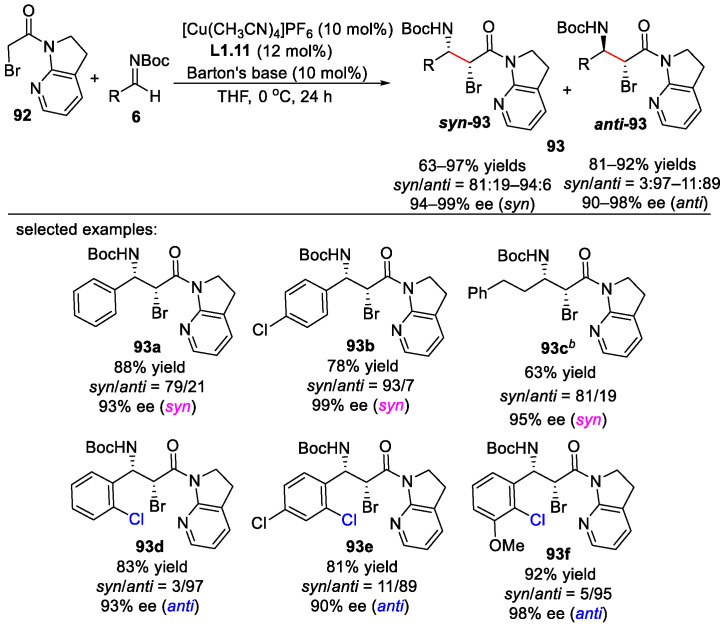
Cu(I)-catalyzed asymmetric Mannich of amide **92** with imines.

**Scheme 45:**
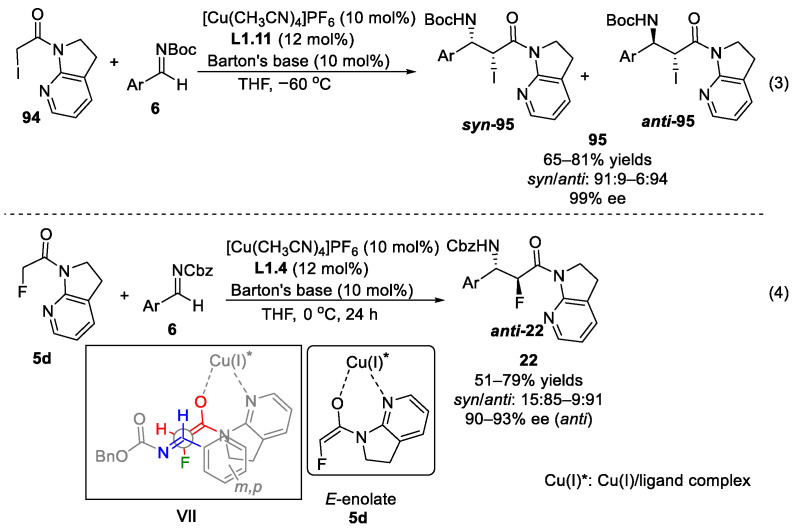
Cu(I)-catalyzed asymmetric Mannich of *α*-I 7-azaindoline amide and *α*-F 7-azaindoline amide with imines.

The Cu(I)/**L1.11**/Barton’s base catalyst was used in the Mannich reaction between *α*-Br 7-azaindoline amide **92** and imines **6**, which provided adducts **93** in good yields with excellent enantioselectivities. The diastereoselectivity of products **93** showed a similar trend to that of products **90** (Scheme 44). The *α*-I 7-azaindoline amide **94** was used as a competent latent enolate to react with aromatic imines **6** for the asymmetric synthesis of *α*-I *β*-amino acid derivatives **95** (Scheme 45 Formula (3)). The diastereoselectivity trend observed in Scheme 42 was also shown for *α*-I 7-azaindoline amide **94**. The authors previously reported the transformation between *α*-F 7-azaindoline amide **5d** and *N*-Cbz imines **6b** catalyzed by the [Cu(CH_3_CN)_4_]PF_6_/**L1.4**/Barton’s base, in which Mannich adducts **22** were received in 51–79% yield with 15:85–9:91 (*syn*/*anti*) dr and 90–93% ee (Scheme 45 Formula (4)). In the specific case of *α*-F 7-azaindoline amide **5d**, the reaction proceeded via the *E*-enolate, which benefited from the smaller steric factor and smaller dipole moment in the fluorine-substituted case. However, in the cases of *α*-Cl 7-azaindoline amide **91**, *α*-Br 7-azaindoline amide **92**, and *α*-I 7-azaindoline amide **94**, the reaction occurred via the *Z*-enolate, meaning that the observed absolute configuration at the *α*-position was opposite to that of *α*-F 7-azaindoline amide **5d**. The *E*-enolization intermediate of the *α*-F 7-azaindoline amide **5d** case reacted with imines **6,** bearing *m*- or *p*- substituents, following the transition state model **VII**, to provide products **22** with high *anti*-selectivity.

### 2.6. α-Oxygen-Substituted 7-Azaindoline Amide as Nucleophiles

**Scheme 46:**
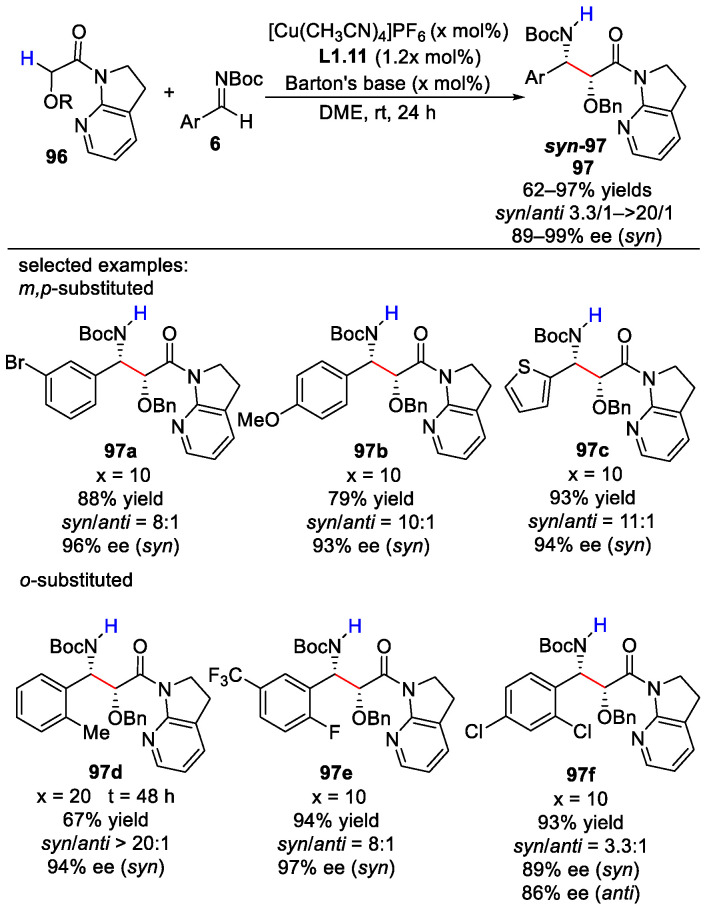
Cu(I)-catalyzed asymmetric Mannich of *α*-oxygen-substituted 7-azaindoline amide with imines.

**Scheme 47:**
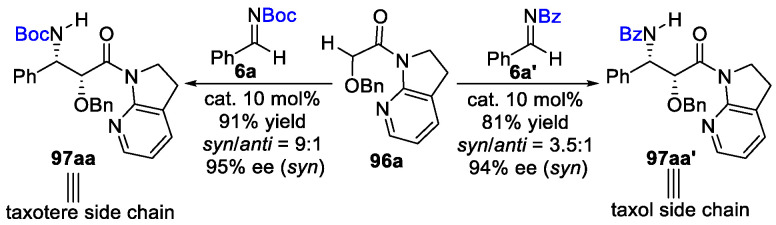
Asymmetric Mannich reaction of *α*-oxygen-substituted 7-azaindoline amide with imines.

Encouraged by the successful developments of the Mannich reaction with *α*-substituted (*α*-thio-, fluoroalkyl-, nitrogen-, alkyl-, halo-) 7-azaindoline amide, Shibasaki and co-workers explored a direct catalytic asymmetric reaction between *α*-oxygen-substituted 7-azaindoline amide **96** and imines **6** catalyzed by the [Cu(CH_3_CN)_4_]PF_6_/**L1.11**/Barton’s base (Scheme 46) [88]. In this process, a series of *α*-hydroxy-*β*-amino carboxylic acid derivatives **97** were obtained with good yields (up to 97%) and moderate to high stereoselectivities (3.3:1→20:1 (*syn*/*anti*)), with excellent enantioselectivities (up to 99% ee). The *m′*- or *p′*-substituted *o*-halogen imines were partially tolerated and the configuration of **97e**–**f** was influenced by the *m′*- or *p′*-substituent. More importantly, *α*-OBn 7-azaindoline amide **96a** reacted with *N*-Boc imine **6a** and *N*-Bz imine **6a′** to construct the side chains of Taxol and Taxotere, respectively (Scheme 47).

**Scheme 48:**
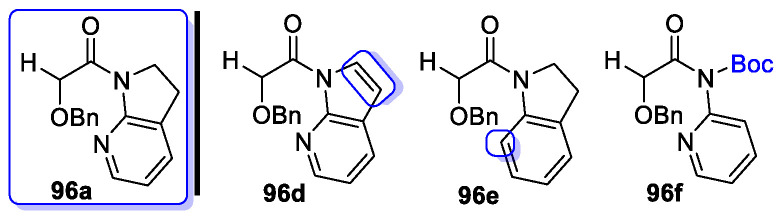
Structurally related amides failed in the Mannich reaction.

The results of control experiments showed that *α*-oxygen-substituted amides **96a**–**c** reacted with imines, whereas amides **96d**–**f**, which had a similar structure to **96a**, did not react with imines, indicating that the 7-azazindoline group was the optimal structure to improve the reaction activity and stereoselectivity (Scheme 48).

**Scheme 49:**
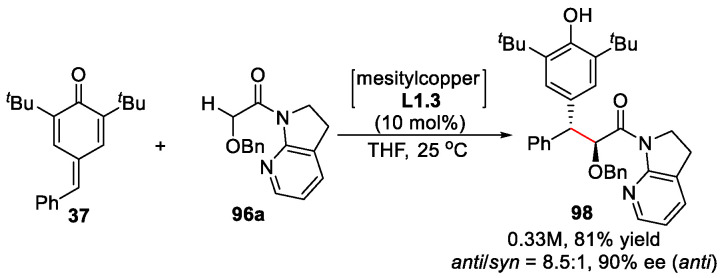
Asymmetric 1,6-conjugate addition of *α*-OBn 7-azaindoline amide and *p*-QMs.

In 2018, Shibasaki’s group also reported a transformation between *α*-OBn 7-azaindoline amide **96a** and *p*-QMs **37,** catalyzed by the mesitylcopper/**L1.3** complex via the asymmetric 1,6-conjugate addition, providing the corresponding adduct **98** with 81% yield with 8.5:1 (*anti*/*syn*) dr and 90% ee (Scheme 49) [78].

**Scheme 50:**
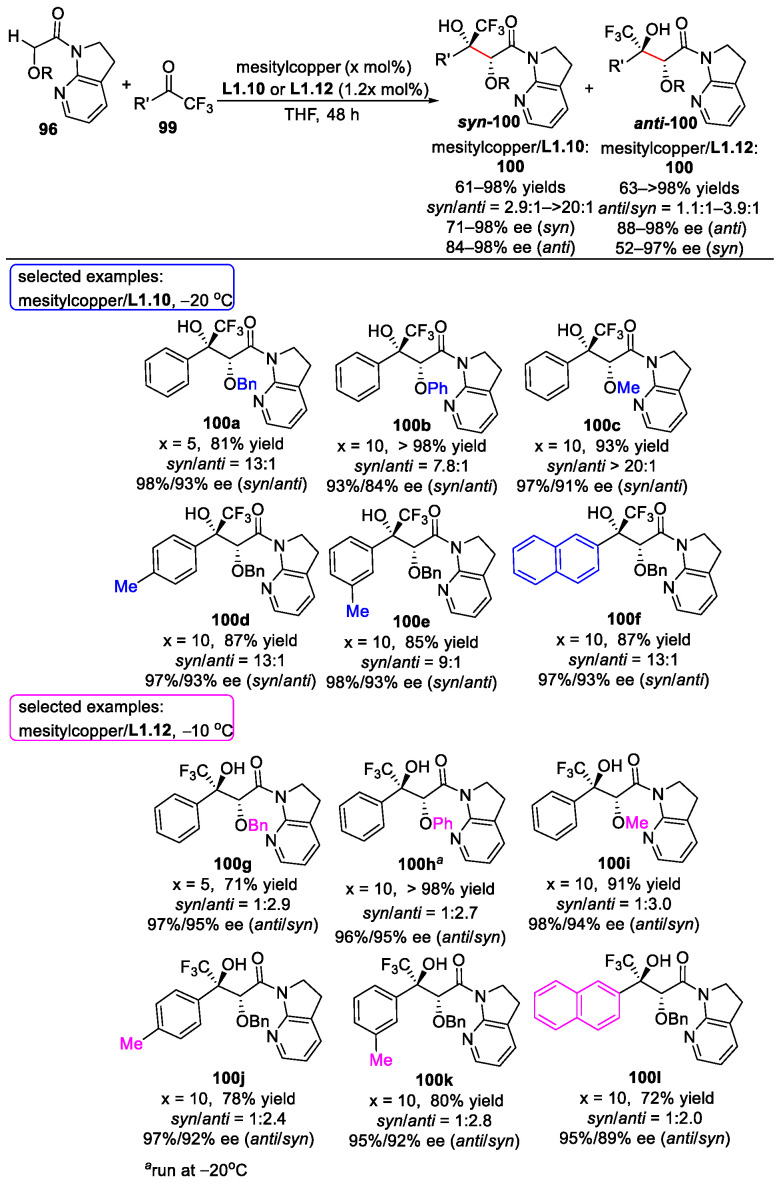
Asymmetric aldol reaction of *α*-OR 7-azaindoline amide with *α*-CF_3_ ketones.

**Scheme 51:**
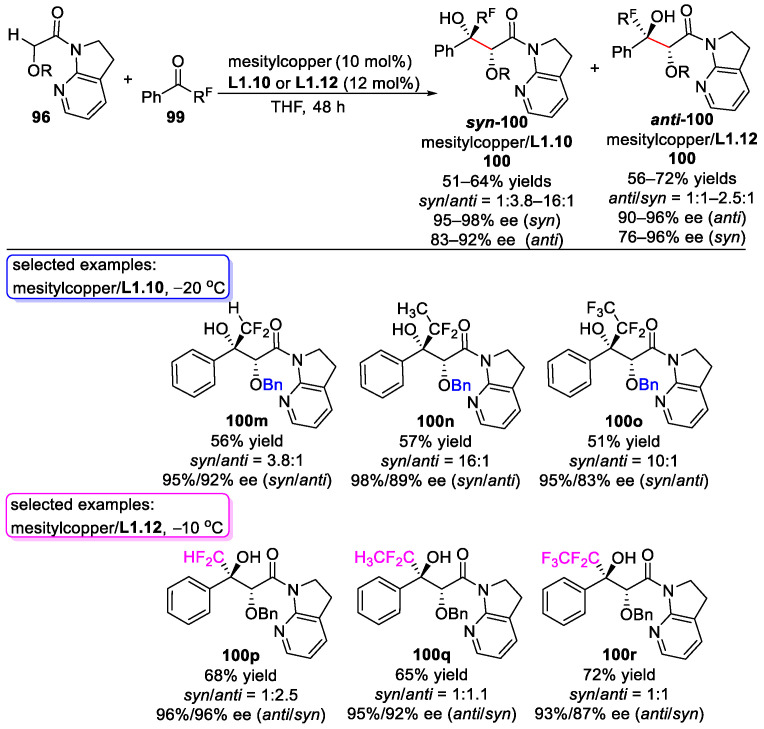
Asymmetric aldol reaction of *α*-OBn 7-azaindoline amide to *α*-fluorinated ketones.

In their ongoing project to simplify the synthesis of a variety of fluorinated compounds, Shibasaki and co-workers developed a direct catalytic asymmetric aldol reaction of *α*-oxygen-substituted 7-azaindoline amide **96** with *α*-fluorinated ketones **99**. All the involved *α*-alkoxy substituents, *α*-OBn, -OPh, -OMe, -OPMB, -Oallyl, -OMOM, OBOM amides, were tolerated to afford 1,2-dihydroxycarboxylic acid derivatives **100** in a highly stereoselective manner (Scheme 50) [89]. The diastereoselectivity switched smoothly, depending on the type of the chiral ligands, which was attributed to the reaction occurring through an open transition state. The *syn*-selectivity of adduct **100a** was not affected by *α*-OBn 7-azaindoline amide **96a** in the gram reaction with the mesitylcopper/**L1.10** complex as a catalyst. Other *α*-alkoxy substituted amides, such as *α*-OPh and *α*-OMe amides, were also suitable for this catalytic system, and the corresponding products **100b**–**c** were obtained with *syn*-selectivity. The *α*-alkoxy substituted 7-azaindoline amides **96a**–**c** reacted with *α*-fluorinated ketones **99** to produce the desired products *anti*-**100g**-**i** by switching the ligand **L1.10** to **L1.12** of the current catalytic system. The structure of the ketones influenced the activity and diastereoselectivity, probably due to the open transition state. The *o-*substituted aryl ketones did not react with *α*-alkoxy substituted 7-azaindoline amides, while the *p*- or *m*-substituted ketones readily and smoothly reacted with *α*-OBn 7-azaindoline amide **96a**. The major isomers of products **100d**–**f** with *syn*-configuration were obtained using ligand **1.10**, and products **100j**–**l** with the *anti*-configuration were obtained using the ligand **1.12**. Other fluoroalkyl-substituted ketones **99p**–**r** were also explored. Notably, difluoromethyl ketone reacted with *α*-OBn 7-azaindoline amide **96a** povided the major isomers of product **100m** with *syn*-configuration instead of *anti*-configuration, using mesitylcopper/**L1.10** complex as a catalyst, probably because the the CF_2_H group has a hydrogen bonding capability, which influenced the reaction face attacked by Cu(I) enolate intermediate **101** (Scheme 51).

**Scheme 52:**
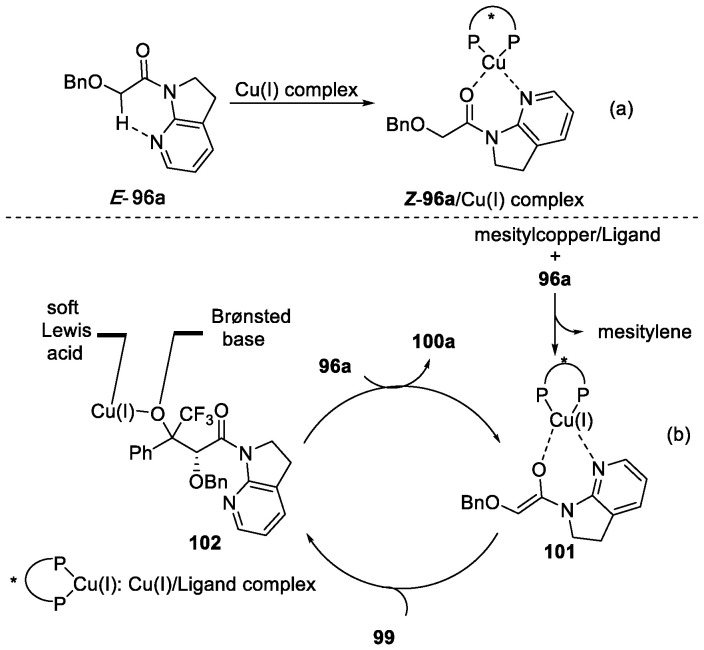
(**a**) The (*E*)-*α*-OBn amide bidentate coordination to Cu(I) complex; (**b**) a possible reaction pathway for the aldol reaction of *α*-OBn 7-azaindoline amide and α-fluorinated ketones.

Similar to the previously reported *α*-substituted amides, the (*E*)-*α*-OBn 7-azaindoline amide **96a** was coordinated with the Cu(I) complex to obtain the (*Z*)-*α*-OBn 7-azaindoline amide/Cu(I) complex (Scheme 52a). A possible aldol process proposed by the authors, Cu(I) enolate complex **101,** was formed by irreversible deprotonation, and then the stereoselective aldol reaction was carried out with the *α*-fluorinated ketone **99** as the electrophile, affording the Cu(I) aldolate intermediate **102**. Intermediate **102** acted as a soft Lewis acid/Brønsted base cooperative catalyst to promote the deprotonation of *α*-OBn 7-azaindoline amide **96a** and the subsequent catalytic cycle process (Scheme 52b).

## 3. α,β-Unsaturated 7-Azaindoline Amides Act as Electrophiles

Conjugate addition is beneficial for electron-deficient alkenes and the nucleophiles to form larger molecular scaffolds. In general, the reaction efficiency is largely determined by the electrophilicity of the electron-deficient alkenes, and relevant substrates include *α,β*-unsaturated ketones, unsaturated aldehydes and nitroolefins, which have been widely reported. *α,β*-unsaturated carboxylic acids are relatively less explored due to their weak electrophilicity, although the products added to these compounds are of high synthetic value. In recent years, the catalytic asymmetric reaction using *α,β*-unsaturated 7-azindolinolamide as an electrophile has become a very interesting topic and was studied by Shibasaki, Yuan and Wu’s group.

The *α*-substituted 7-azindoline amides summarized above are easily enolized via complexation with metal complexes, and this activation pattern also applies to the electrophilic activation of *α,β*-unsaturated 7-azindoline amides. X-ray crystallographic analysis shows that *α,β*-unsaturated 7-azindoline amides tend to be in the *E*-conformation, their conversion to the *Z*-conformation is achieved by adding Cu(I) complexes, and the *β*-carbon electrophilicity of *α,β*-unsaturated 7-azindoline amides in the *Z*-conformation is increased.

### 3.1. α,β-Unsaturated 7-Azaindoline Amides as Electrophiles in Vinylogous Conjugate Addition

**Scheme 53:**
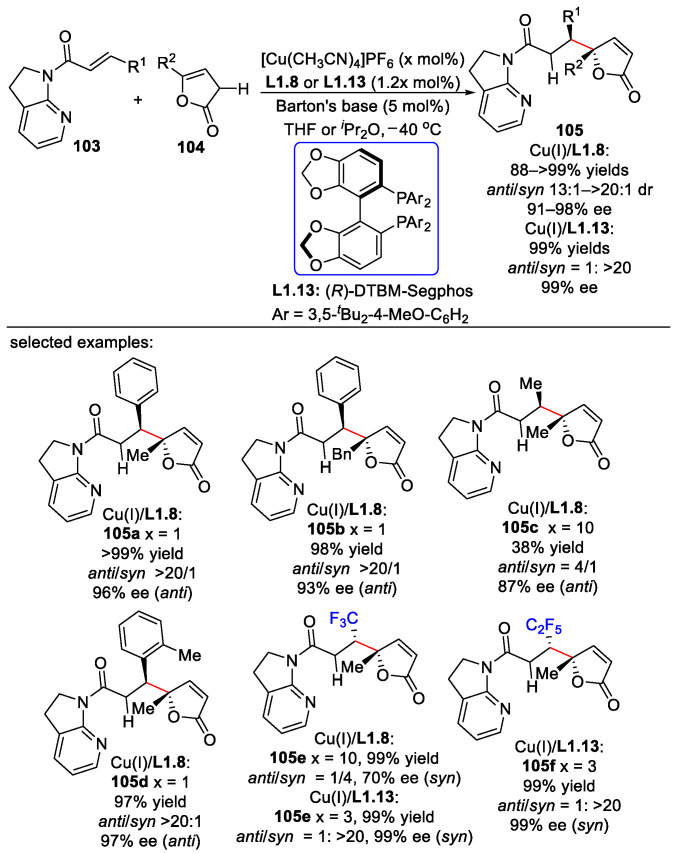
Asymmetric vinylogous conjugate addition of *α,β*-unsaturated 7-azaindoline amides with γ-butyrolactones.

**Scheme 54:**
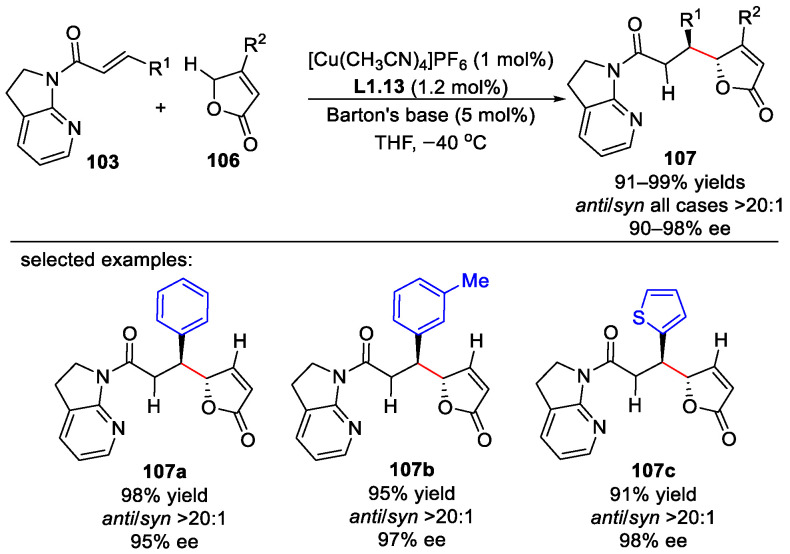
Asymmetric vinylogous conjugate addition of *α,β*-unsaturated 7-azaindoline amides with *α,β*-unsaturated *γ*-butyrolactones.

In 2016, Shibasaki and co-workers first reported an asymmetric conjugate addition reaction of *α,β*-unsaturated 7-azaindoline amides **103** with *γ*-butyrolactones **104** using the [Cu(CH_3_CN)_4_]PF_6_/**L1.8** as a cooperative catalyst, providing the desired conjugate adducts **105** in 88→99% yield with 13:1→20:1 (*anti*/*syn*)) dr and 91–98% ee. When the [Cu(CH_3_CN)_4_]PF_6_/(*R*)-DTBM-Segphos **L1.13** was used, the reaction of *β*-CF_3_-substituted and *β*-C_2_F_5_-substituted 7-azaindoline amides with *γ*-butyrolactones produced the desired products **105g**-**h** with high yield and excellent stereoselectivities using *i*Pr_2_O as the solvent (Scheme 53) [90]. The authors also explored the asymmetric vinylogous conjugate addition of *α,β*-unsaturated *γ*-butyrolactones **106** and *α,β*-unsaturated 7-azaindoline amides **103** catalyzed by the Cu(I)/**L1.13** complex, which provided access to desired products **107,** bearing continuous trisubstituted stereogenic centers with high yield and excellent stereoselectivities (Scheme 54).

**Scheme 55:**
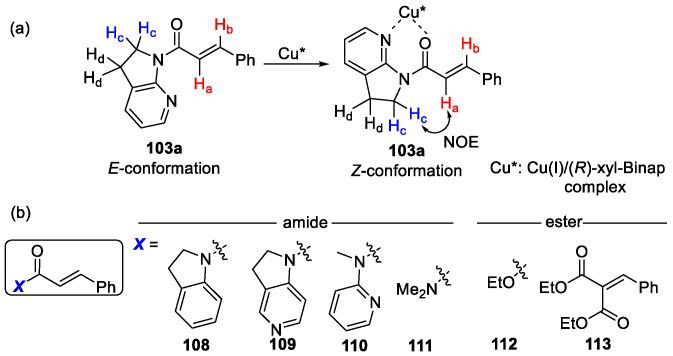
(**a**) The (*E*)-*α,β*-unsaturated 7-azaindoline amide bidentate coordination to the Cu(I) complex; (**b**) the use of structurally similar amides as electrophiles failed in the reaction.

As shown in Scheme 55a, the *α,β*-unsaturated 7-azaindoline amide **103** with an *E*-configuration were converted to the *Z*-configuration after coordination with the Cu(I)/**L1.8** complex, as observed in the NMR study. This coordination resulted in an increase in the electrophilicity of the *β*-carbon of the *α,β*-unsaturated 7-azaindoline amide **103**. Using the Cu(I)/**L1.8** complex as a catalyst, a series of amides **108**–**111**, which have a similar structure to 7-azindolinamide, were not suitable for the catalytic system and did not react with *γ*-butyrolactones. Moreover, esters **112**–**113** failed in this reaction system (Scheme 55b). These results indicated that 7-azazindoline was the best directing group in terms of reactivity and stereoselectivity.

### 3.2. α,β-Unsaturated 7-Azaindoline Amides as Electrophiles in 1,3-Dipolar Cycloaddition

**Scheme 56:**
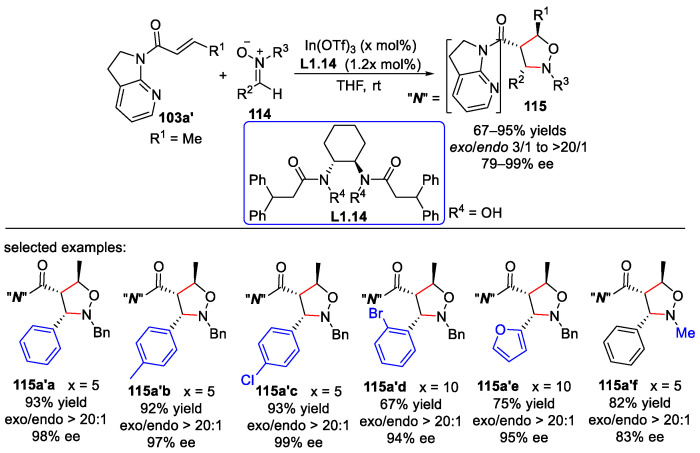
Asymmetric 1,3-dipolar cycloaddition of *α,β*-unsaturated 7-azaindoline amides with aromatic nitrones.

**Scheme 57:**
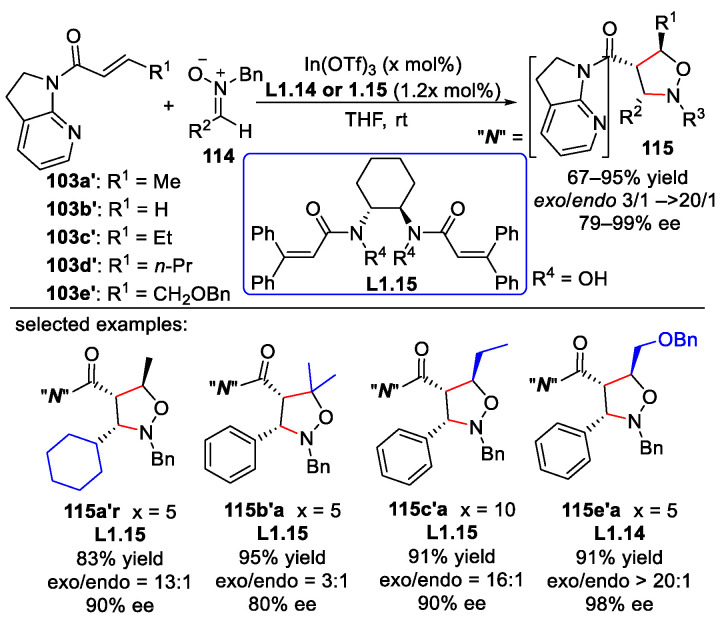
Asymmetric 1,3-dipolar cycloaddition of *α,β*-unsaturated 7-azaindoline amides with aliphatic nitrones.

To further illustrate the important role of the 7-azazindoline group of *α*,*β*-unsaturated amides **103** in controlling the stereoselectivity of the reaction, in 2017, Shibasaki and co-workers subsequently examined the catalytic asymmetric synthesis of isoxazolidines **115** via the asymmetric *exo*-selective 1,3-dipolar cycloaddition of *β*-alkyl *α,β*-unsaturated amides **103a′** and aromatic nitrones **114** under In(III) complex catalysis (Scheme 56) [91]. The use of 5 mol% or 10 mol% of In(OTf)_3_/bishydroxamic acid complex promoted a smooth reaction at room temperature to afford highly substituted isoxazolidines **115** in 67–95% yields with 3:1→20:1 (*exo*/*endo*) dr and 79–99% ee (*exo*). The catalytic system has a wide range of substrate universality. The *o*-Br-substituted aromatic nitrone **114d** also reacted with **103a′** for the asymmetric synthesis of isoxazolidines **115a′d**, where 10 mol% of the In(OTf)_3_/**L1.14** catalyst was required due to the steric hindrance factor of **114d**. The authors expanded the range of *β*-alkyl *α,β*-unsaturated amides with aliphatic nitrones using the In(III) complex catalyst (Scheme 57).

**Scheme 58:**
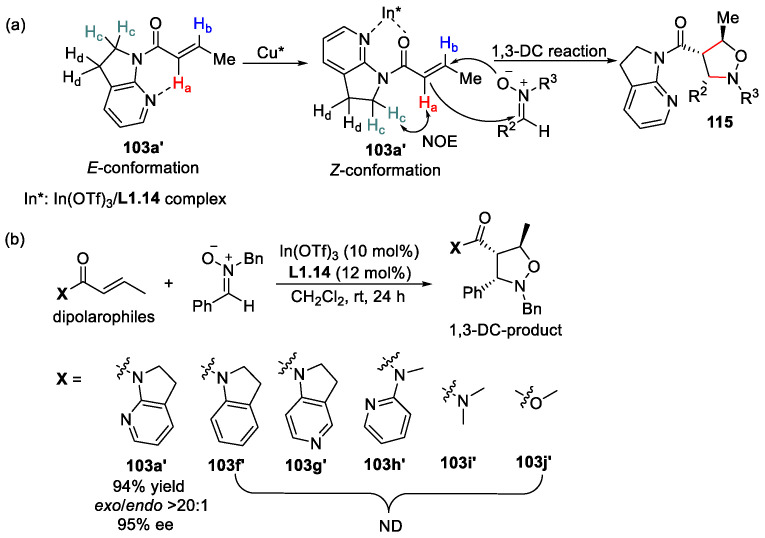
(**a**) ^1^H NMR analysis of amide **103a′** and amide **103a′** binding to In(III)/**L1.14** complex; (**b**) the control experiment of structurally similar amides with aromatic nitrones.

NMR analysis shows that an intramolecular hydrogen bond is formed between the nitrogen atom of the pyridinyl group and the *α*-hydrogen atom. This finding confirms that amide **103a′** contains an *E*-configuration in the solution. After coordinating the amide **103a′** with In(III)/**L1.14**, NOE analysis showed that the amide **103a′** was converted to a *Z*-configuration (Scheme 58a). Subsequently, a 1,3-dipole cycloaddition reaction occurred between *Z*-amide **103a′** and nitrones **114**. A series of similarly structured amides **103f′**–**i′** and methyl esters **103j′** were investigated to illustrate the important role of 7-azazindoline in reactivity and stereoselectivity (Scheme 58b). It was found that none of these amides reacted with nitrone **114a** in the current catalytic system. Indolinamides **103f′** and 5-azazindolinamides **103g′** did not react with nitrone, suggesting that the presence of a nitrogen atom at the 7-position is essential for the reaction to proceed and that the substrates and In(III) complexes most likely interact via bidentate coordination. The importance of the five-membered pyrrole scaffold in 7-azazindoline was further demonstrated by the failure of amide **103h′** in the reaction.

**Scheme 59:**
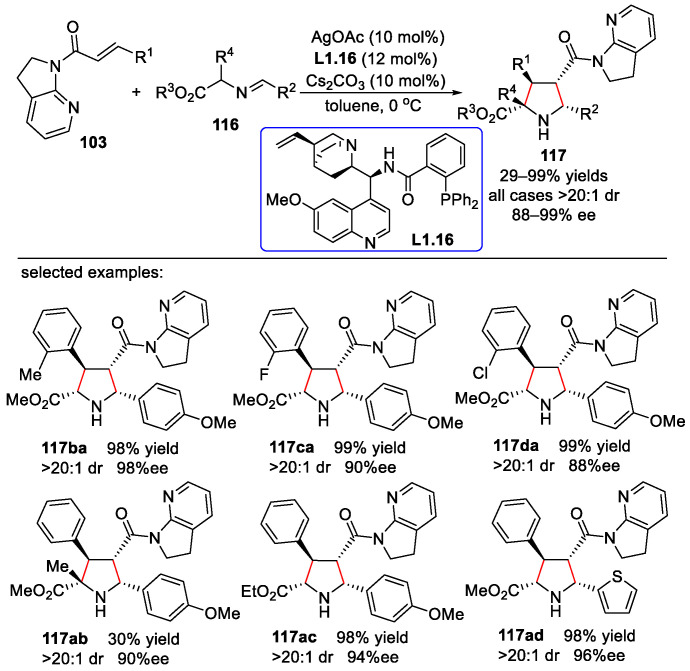
Asymmetric 1,3-dipolar cycloaddition of *α,β*-unsaturated 7-azaindoline amides with azomethine ylides.

Inspired by the fact that *α,β*-unsaturated 7-azaindoline amides were highly efficient electrophiles in the construction of isoxazolidines, and based on the important role of the 7-azindoline group of the amides in controlling the stereoselectivity of the reaction, Yuan’s group developed a highly enantio- and diastereoselective 1,3-dipolar cycloaddition between *α,β*-unsaturated 7-azaindoline amides **103** and azomethine ylides **116** catalyzed by the AgOAc/quinine-derived aminophosphine complex (Dixon’s catalyst) in 2019 (Scheme 59) [92]. In this reaction, highly substituted pyrrolidine derivatives **117** bearing four contiguous stereogenic centers were obtained in 29–99% yields with >20:1 dr and 82–99% ee using aromatic, heteroaromatic and aliphatic *α,β*-unsaturated 7-azaindoline amides as electrophiles. It was noteworthy that *α,β*-unsaturated 7-azaindoline amides **103** with electron-donating groups exhibited much higher ee values than those with electron-withdrawing groups in the 1,3-dipolar cycloaddition. The reaction of *α*-methyl azomethine ylide **117b** proceeded with a dramatic decrease in yield (30%), which was attributed to the greater steric hindrance at the R^4^ position in the azomethine ylides **116b**.

**Scheme 60:**
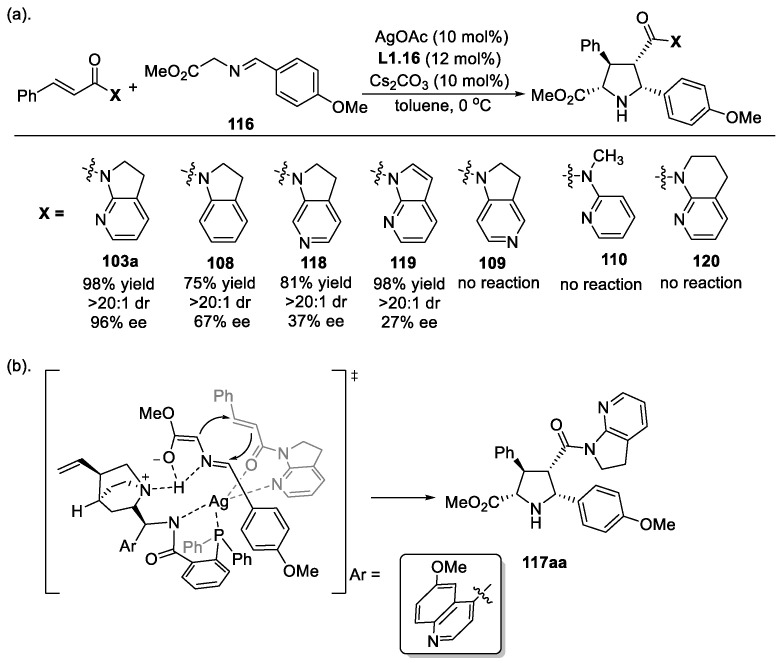
(**a**) The reaction results of structurally similar amides with azomethine ylides; (**b**) a possible reaction transition state.

Yuan’s group also investigated the role of the 7-azazindoline moiety in *α,β*-unsaturated amides. A series of amides of similar structure reacted with azomethine ylides **116a** under the present catalytic conditions, as follows: Indolinylamide **108** was suitable for the reaction and provided the target product with 75% yield, with >20:1 dr and 67% ee. The reaction of 6-azazindoline amide **118** and 7-azaindole amide **119** with **116a** produced the corresponding products, with 37% ee and 27% ee, respectively. However, other amides, such as **109**, **110** and **120,** failed following 1,3-dipolar cycloaddition (Scheme 60a). The results of these control experiments showed that 7-azazindoline was the optimal structure for controlling the stereoselectivity of the reaction. According to the results of the control experiments presented above, the possible reaction transition state for the 1,3-dipolar cycloaddition reaction of *α,β*-unsaturated 7-azazindoline amides **103** and azomethine ylides **116** was proposed (Scheme 60b). First, AgOAc coordinates with the aminophosphine ligand **L1.16**, which is derived from quinine, to form the complex. The AgOAc/**L1.16** complex activated amide **103** by coordinating Ag^+^ with the pyridinyl nitrogen atoms and carbonyl oxygen atoms of the 7-azindolinyl moiety in the amide. The methylene group of the azomethine ylide **116** is deprotonated by the tertiary amine in the AgOAc/**L1.16** complex to form an anion. The **116** was activated through hydrogen bonding, which was then followed by a 1,3-dipolar cycloaddition reaction with the activated amide. This reaction resulted in the formation of enantioenriched pyrrolidine derivatives **117**.

**Scheme 61:**
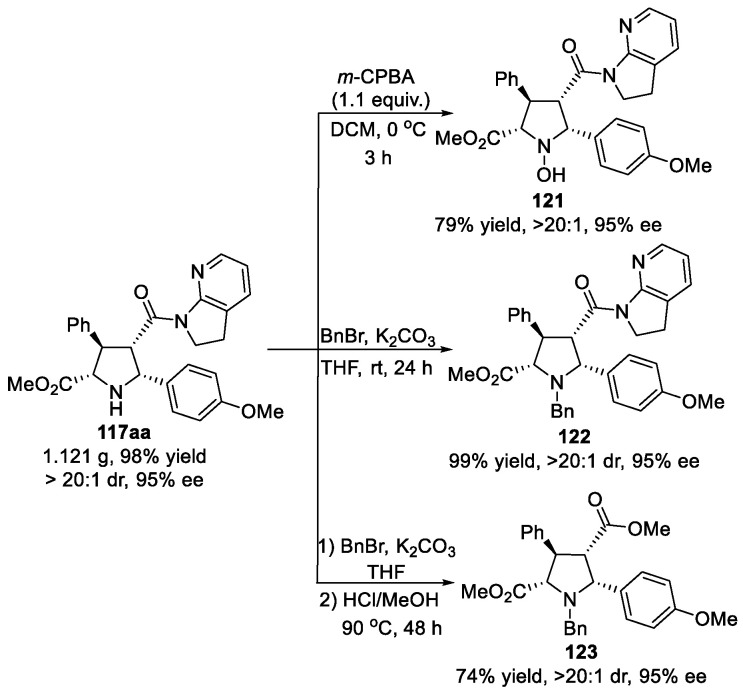
Transformations of the adduct **117aa**.

To demonstrate the synthetic utility of highly substituted pyrrolidine derivatives, transformations of the pyrrolidines **117aa** were also investigated (Scheme 61). The treatment of **117aa** with *m*-chloroperoxybenzoic acid did not cause a decrease in stereoselectivity for the *N*-hydroxyl pyrrolidine **121**. Notably, the desired carboxylic ester derivative **123** was smoothly obtained with 74% yield with >20:1 dr and 95% ee by first removing 7-azindoline from product **122** under acidic conditions, followed by esterification.

**Scheme 62:**
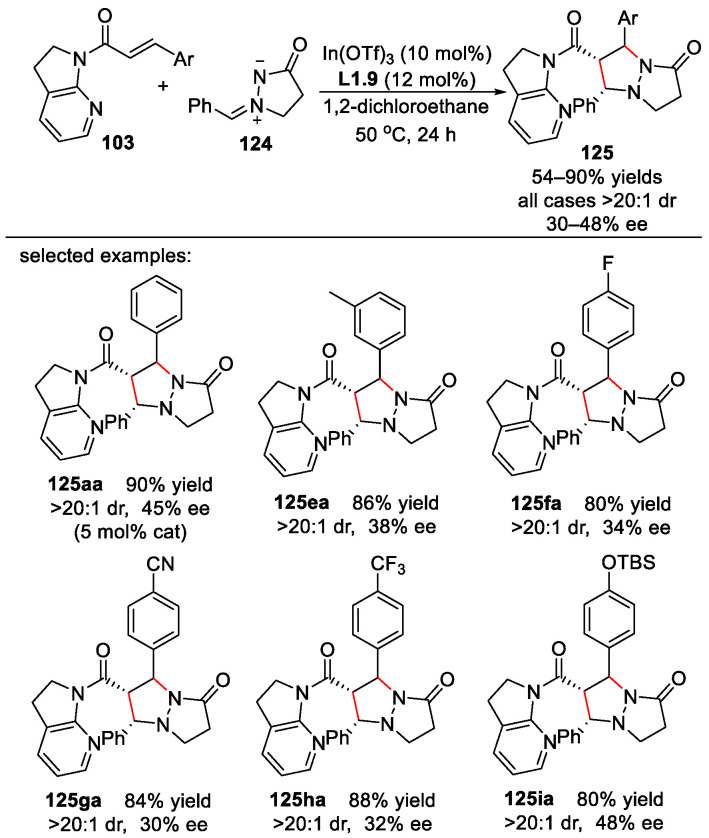
Asymmetric 1,3-dipolar cycloaddition of *α,β*-unsaturated amides and azomethine imine.

**Scheme 63:**
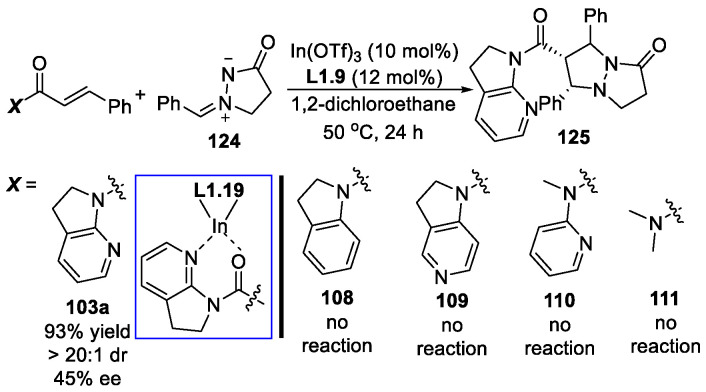
The control experiments of structurally similar amides with azomethine imine.

In 2020, Shibasaki and colleagues investigated the asymmetric 1,3-dipolar cycloaddition of *α,β*-unsaturated 7-azaindoline amides **103** and azomethine imines **124** using the In(OTf)_3_/bishydroxamic acid **L1.9** complex as a catalyst, affording bicyclic compounds **125** with 54–90% yield in all cases >20:1 dr and 30–48% ee (Scheme 62) [93]. The *p*-OTBS-substituted *α,β*-unsaturated 7-azaindoline amides provided the corresponding product **125ia** with 48% ee, which is higher than that of the amides substituted with electron-withdrawing groups. In the current catalytic system, structurally similar amides **108**–**111** did not react with azomethine imine **124**, indicating that the 7-azazindoline moiety of amide **103** is crucial for activating the amides and enhancing the stereoselectivity of the target products (Scheme 63).

### 3.3. α,β-Unsaturated 7-Azaindoline Amides Act as Electrophiles in Michael/Aldol Cascade Reaction

**Scheme 64:**
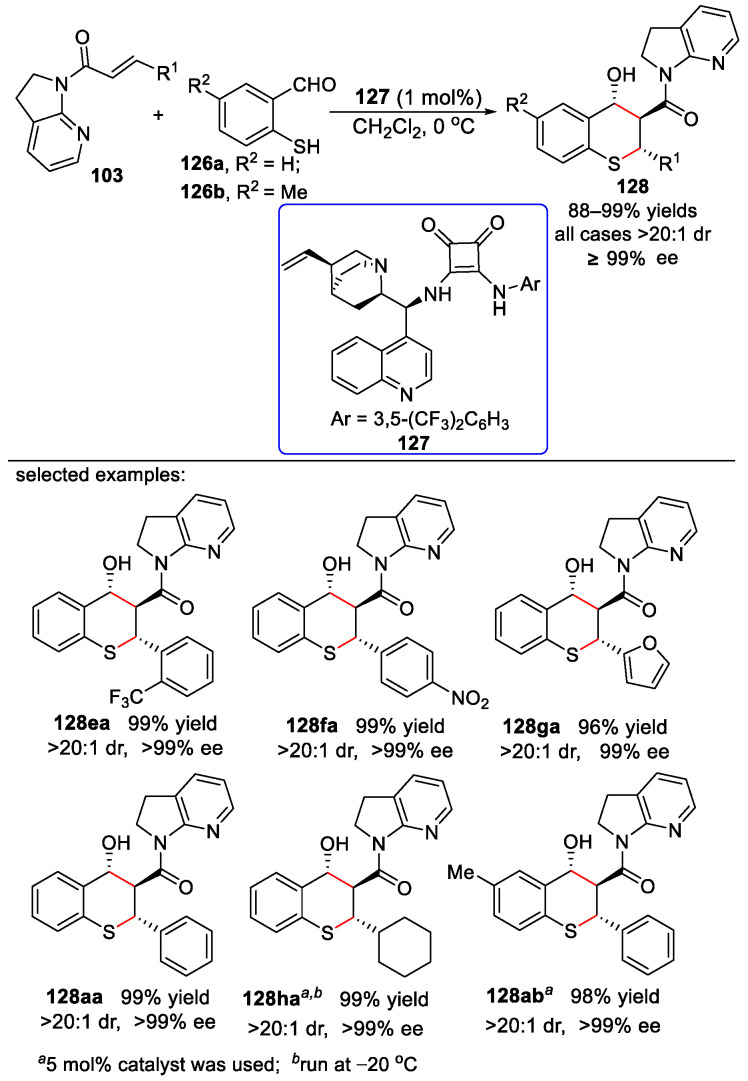
Asymmetric Michael/aldol cascade reaction of *α,β*-unsaturated amides and 2-mercaptobenzaldehyde.

The first example of the organocatalyzed asymmetric Michael/aldol cascade reaction with *α,β*-unsaturated 7-azaindoline amides as electrophilic partners was reported by Yuan’s group in 2019, as shown in Scheme 64 [94]. The process was catalyzed by only 1 mol% of cinchonidine-derived bifunctional squaramide **127**, in which *α,β*-unsaturated 7-azaindoline amides **103** underwent an enantioselective Michael/aldol reaction with the 2-mercaptobenzaldehyde **126**. The product thiochromenes **128,** bearing three contiguous stereogenic centers, were afforded with 88–99% yield, >20:1 dr and ≥99% ee. Both *α,β*-unsaturated 7-azaindoline amides substituted with electron-donating groups and electron-withdrawing groups were able to effectively participate in the reaction, demonstrating the broad substrate universality of this reaction. A cyclohexyl-substituted substrate was investigated and the target product **128ha** was produced with a high yield and stereoselectivity at −20 °C using 5 mol% catalyst loadings. Notably, a methyl substituent on the C5 position of the phenyl group of 2-mercaptobenzaldehyde **126b** was also compatible with the current catalytic system. The desired product, **128ab,** was successfully obtained using a 5 mol% catalyst **127**.

**Scheme 65:**
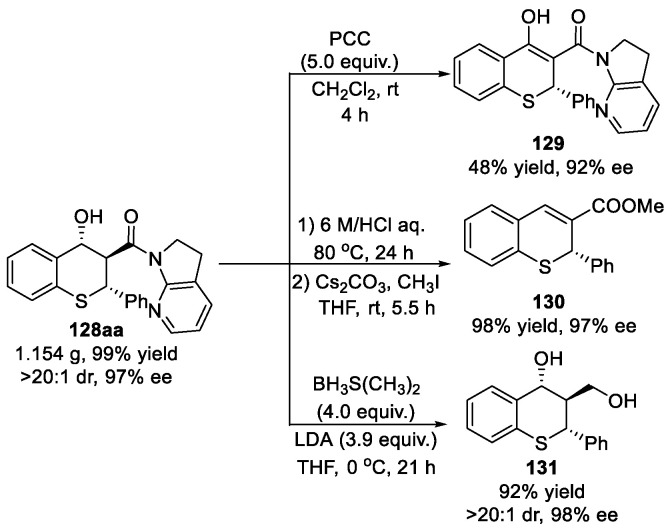
Different transformations of the product **128aa**.

**Scheme 66:**
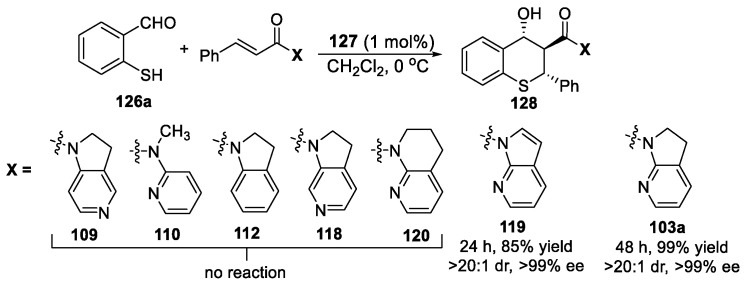
The results of control experiments with structurally similar amides and 2-mercaptobenzaldehyde.

The potential application value of this methodology was confirmed by the gram reaction and diversity transformation experiments (Scheme 65). To investigate the important role of the 7-azazindoline group of *α,β*-unsaturated amides in terms of their reactivity and stereoselectivity, a series of amides with similar structure were reacted with 2-mercaptobenzaldehyde (Scheme 66). The failure of amides **109**, **112** and **118** to react with 2-mercaptobenzaldehyde indicated that the position of the nitrogen atom in the pyridine ring of 7-azazindoline was crucial to the bidentate’s coordination with the organocatalyst. *N*-methyl-(2-pyridyl) amide **110** and 1,2,3,4-tetrahydro-1,8-naphthyridinylamide **120** did not react with 2-mercaptobenzaldehyde, demonstrating that the rigid skeleton of the pyrrole ring of 7-azindoline was also important to the reaction. The *α,β*-unsaturated 7-azaindoline amide **119** was well-tolerated and provided a corresponding product with acceptable results. These control experiments showed that the 7-azazindoline group in amides is the most effective directing group.

**Scheme 67:**
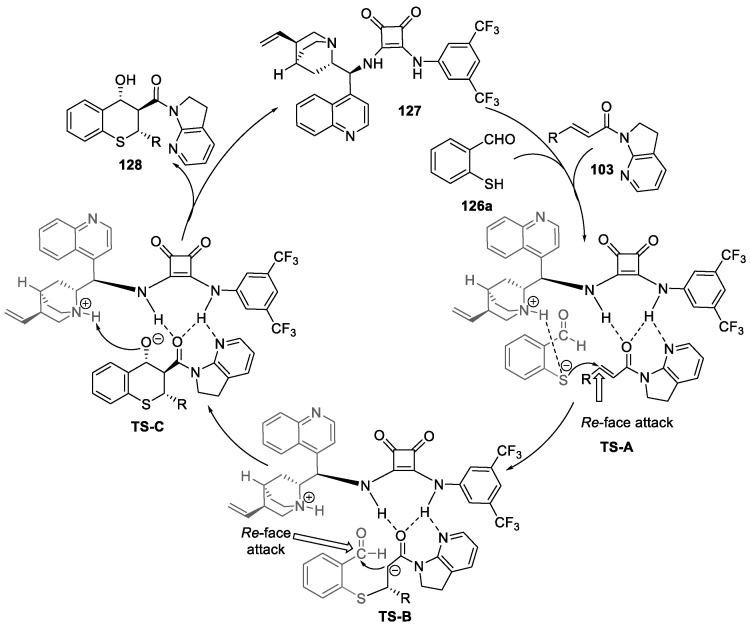
A proposed reaction pathway for organo-catalyzed reaction of *α,β*-unsaturated amides and 2-mercaptobenzaldehyde.

Based on previous reports in the literature [95,96,97,98] and the results of the control experiments, a plausible pathway for the Michael/aldol cascade reaction of *α,β*-unsaturated 7-azazindoline amide and 2-mercaptobenzaldehyde was proposed, as shown in Scheme 67. As presumed, the tertiary amine-squaramide **127** should act in a bifunctional manner. Firstly, the tertiary amine moiety in catalyst **127**, which acts as a base, deprotonated the mercapto group of 2-mercaptobenzaldehyde. At the same time, the squaramide moiety of catalyst **127** activates the *α,β*-unsaturated 7-azazindoline amides **103** through double hydrogen bonds. Secondly, the anion of 2-mercaptobenzaldehyde attacks the *β*-position of *α,β*-unsaturated 7-azazindoline amides from the *Re* face via the sulf-Michael addition reaction (TS-A). Thirdly, the carbanion of the *α,β*-unsaturated 7-azazindoline amides approaches the aldehyde group of 2-mercaptobenzaldehyde from the *Re* face via an intramolecular aldol reaction (TS-B). Finally, the oxygen anion intermediate was protonated to provide the target product **128** (TS-C), and the catalyst **127** was released for the subsequent catalytic cycle.

### 3.4. α,β-Unsaturated 7-Azaindoline Amides Act as Electrophiles in Aminomethylation

**Scheme 68:**
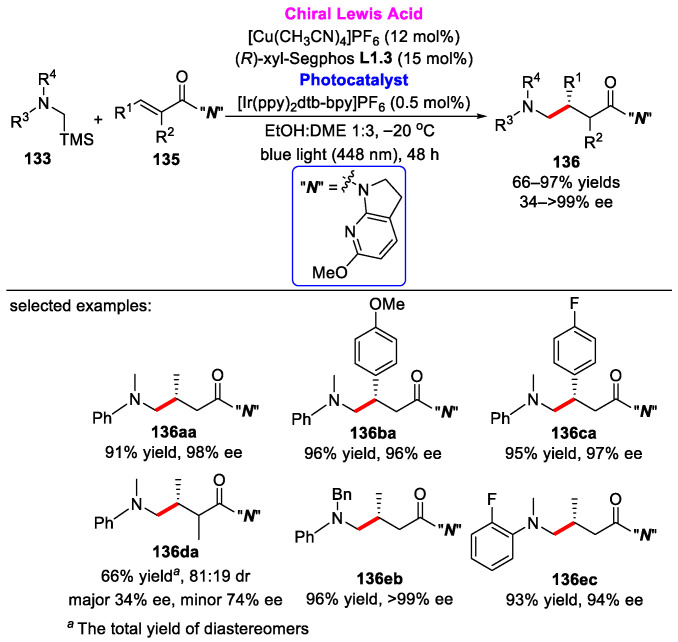
Asymmetric aminomethylation of *α,β*-unsaturated 7-aza-6-MeO-indoline amides and α-silylamines.

**Scheme 69:**
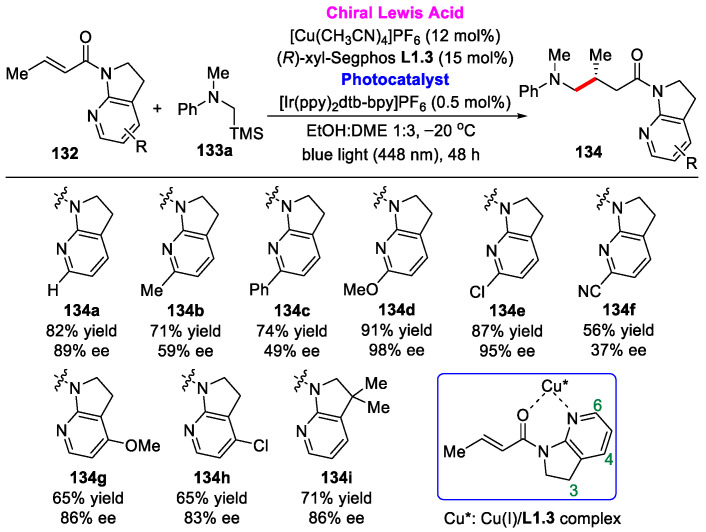
The effect of the substituents of 7-azindoline on the aminomethylation.

Based on the successful example of the Cu-catalyzed 7-azazindoline amide involved in the highly stereoselective construction of a C-C bond via asymmetric reaction and the lack of research on 7-azazindoline as a directing group in the field of photocatalysis, Shibasaki and coworkers developed an enantioselective aminomethylation of *α,β*-unsaturated 7-aza-6-MeO-indoline amides **135** with *α*-silylamines **133** using the Cu(I)/**L1.3** complex and Ir(III) photocatalyst as a synergistic catalyst, producing the *γ*-aminobutyric acid derivatives **136** with 66–97% yield with 88→99% ee (Scheme 68) [99]. After the initial optimization of the reaction conditions, it was found that the target product **134a** was obtained with 89% ee (Scheme 69). The influence of substituents on the 7-azaindoline group in the reaction was then investigated, showing that the activity and enantioselectivity of the reaction were significantly increased when 7-aza-6-methoxy-indoline was used as the directing group. Therefore, 7-aza-6-methoxy-indoline amide was chosen as the electrophile for the aminomethylation with *α*-silylamines. The *α,β*-unsaturated 7-aza-6-MeO-indoline amides **135** with different substituents, independent of the electron-donating or electron-withdrawing groups on the benzene ring, were successfully reacted with *α*-silylamines **133** to provide products **136ba**–**ca** with high yields and high enantioselectivities. The *α*-Me substituted amide **135d** was also tolerated in this reaction, generating the desired product **136da** with a 66% yield with 81:19 dr, and the major isomer with only 34% ee.

**Scheme 70:**
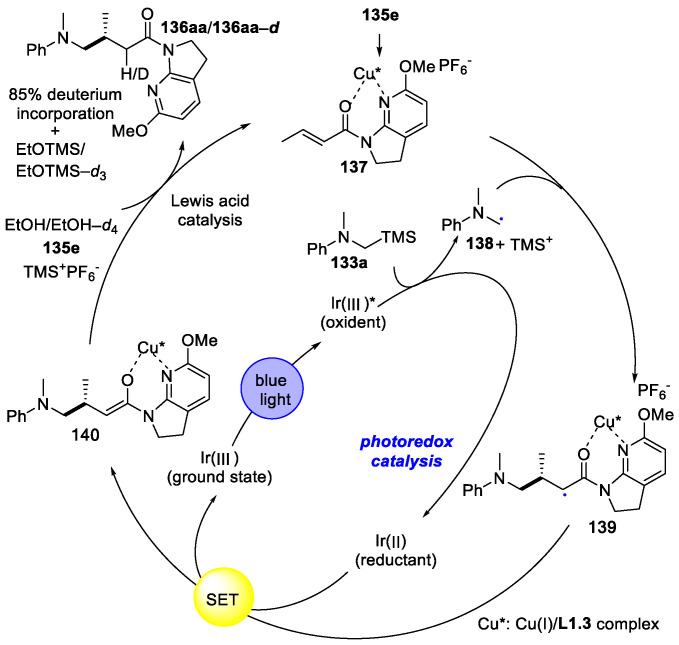
Proposed reaction pathway for Cu(I)/Ir(III) cooperative catalyzed the aminomethylation of *α,β*-unsaturated 7-aza-6-MeO-indoline amides and *α*-silylamines.

A plausible pathway for the aminomethylation of *α,β*-unsaturated 7-aza-6-MeO-indoline amides and *α*-silylamines was proposed, as shown in Scheme 70. The *β*-methyl *α,β*-unsaturated 7-aza-6-MeO-indoline amide **135e** bound to the Cu complex to form complex **137**, which prevented the [2 + 2] photocycloaddition of 7-aza-6-MeO-indoline amide **135e**. The Ir(III) photocatalyst in the ground state was excited to the excited state, and the photoexcited Ir(III)* complex oxidized *α*-silylamine **133a** to produce *α*-amino radical **138** and TMS cations under the condition of blue light irradiation. The 7-aza-6-MeO-indoline amide **135e** in complex **137** was electrophilic enough to couple with the *α*-amino radical **138**, and the resulting *α*-amino radical **139** exhibited good stereoselectivity. Enolate amide **140** was obtained by the single-electron transfer of Ir(II) complex to *α*-amino radical **139**. Meanwhile, the photoexcited Ir(III)* complex returned to the ground state, completing the photocatalytic cycle. Enolate amide **140** is protonated, releasing the *γ*-amino amide **136aa**. When ethanol-*d*_4_ was used instead of regular ethanol, the deuterium-labeled products **136aa**-**d** accounted for 85% of the total yield. This finding further supports the hypothesis that ethanol acts as a source of protons.

**Scheme 71:**
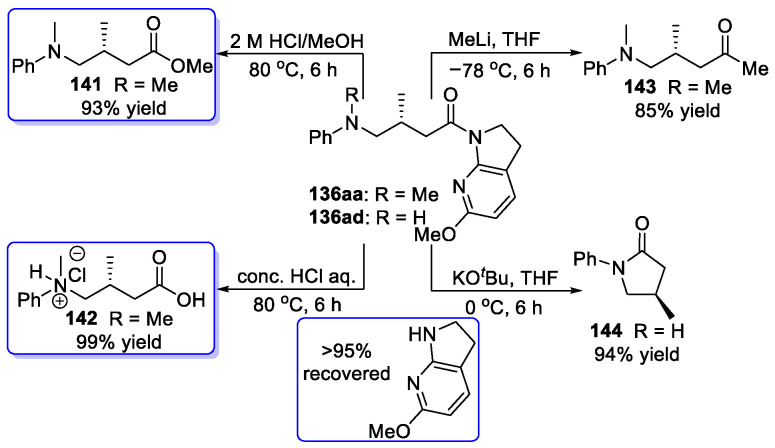
Different transformations of the product **136aa**/**136ad**.

The potential synthetic utility of this method was demonstrated by further transformation of the product (Scheme 71). The treatment of **136aa** with hydrochloric acid provided methyl ester **141** and carboxylic acid **142**, depending on the concentration of hydrochloric acid used. Both **141** and **142** are important derivatives of *γ*-aminobutyric acid, a key structural unit that exists in several drugs used to treat diseases of the central nervous system. Treating **136aa** with an organolithium reagent in tetrahydrofuran at −78 °C, methyl ketone **143** was obtained without over-alkylated product. Lactam **144** was obtained by the simple treatment of product **136ad** with potassium *tert*-butanol. In addition, the 6-methoxy-7-azazindoline was successfully removed, with a recovery of more than 95%.

### 3.5. α,β-Unsaturated 7-Azaindoline Amides Act as Electrophiles in Michael Addition-Initiated Ring-Closure Reaction

**Scheme 72:**
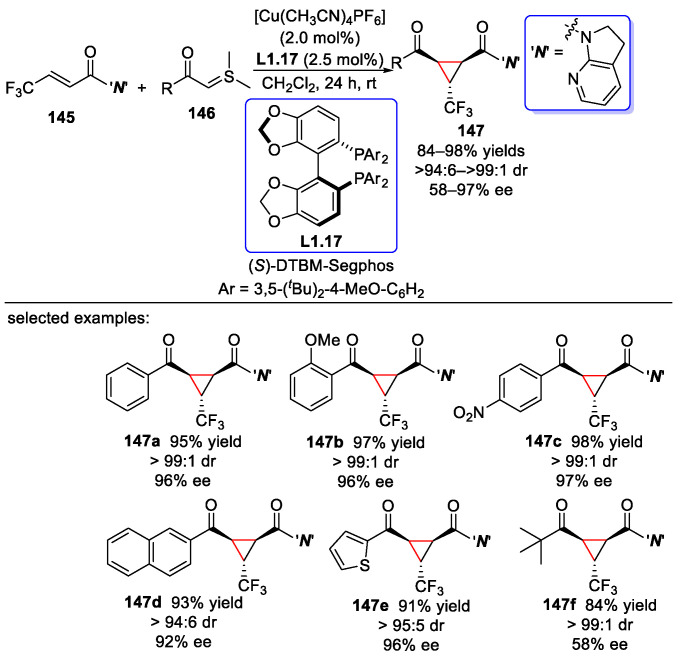
Asymmetric Michael addition-initiated ring-closure reactions of *β*-trifluoromethyl *α,β*-unsaturated 7-azaindoline amides and sulfur ylides.

As part of Shibasaki’s group, ongoing research focuses on asymmetric reactions using *α,β*-unsaturated 7-azaindoline amide as the electrophile under metal catalysis. They developed a highly stereoselective synthesis of 1,2,3-substituted cyclopropanes **147** through the Michael addition-initiated ring-closure reaction of *α,β*-unsaturated 7-azaindoline amides **145** and sulfur ylides **146** catalyzed by chiral Cu(I) complexes (Scheme 72) [100]. In this process, the use of the 2 mol% [Cu(CH_3_CN)_4_]PF_6_/**L1.17** complex smoothly promoted the Michael addition at room temperature to afford CF_3_-functionalized trisubstituted cyclopropanes **147** in 84–98% yields with >94:6→99:1 dr and 58–97% ee. This method was applicable to sulfur ylides with various substituents.

**Scheme 73:**
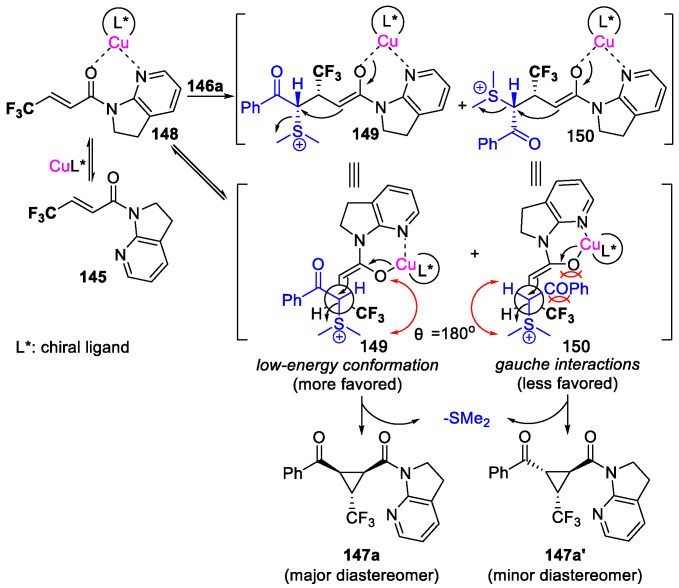
A possible mechanism of *β*-trifluoromethyl *α,β*-unsaturated 7-azaindoline amide and sulfur ylides.

A possible reaction pathway was proposed, as shown in Scheme 73. The *Z*-configuration complex **148** was obtained by the coordination of *β*-CF_3_ *α,β*-unsaturated 7-azaindoline amide **145** and Cu(I)/L* complex, which increased the electrophilicity of amide **145** and facilitated attack by nucleophile **146a**. The major Cu-enolate intermediate **149** was obtained by the formation of C-C bonds at the *β*-position via an irreversible pathway, and then Cu-enolate intermediate **149** underwent intramolecular S_N_2 reaction to provide major diastereomer **147a**. Minor Cu-enolate intermediate **150** provided minor diastereomer **147a′**. The Newman projection shows that major diastereomer **147a** is formed via stable intermediate **149**. This is because the -CF_3_ and the sterically bulkier -COPh group were in the *anti*-periplanar conformation, and the dihedral angle between the Cu-enolate and the -SMe_2_ group was 180°. In contrast, the Cu-enolate, -COPh, and -CF_3_ in intermediate **150** were crowded and underwent a strong torsional interaction. The minor diastereomer **147a′** was probably formed by a high-energy transition state.

**Scheme 74:**
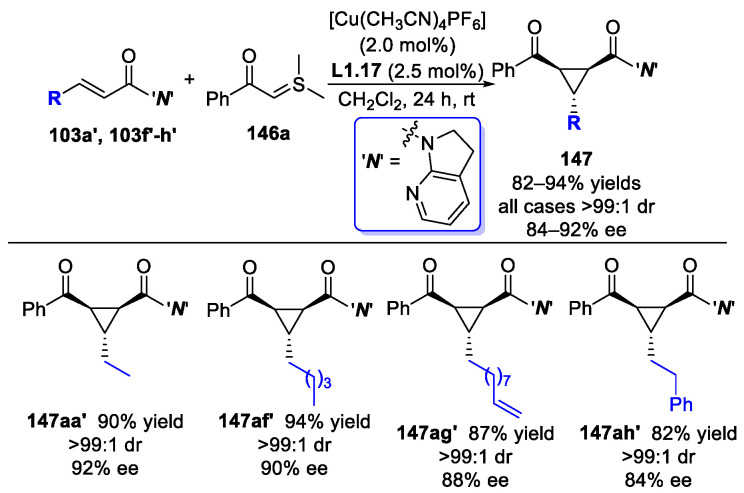
Asymmetric Michael addition-initiated ring-closure reactions of *β*-alkyl *α,β*-unsaturated 7-azaindoline amides and sulfur ylides.

**Scheme 75:**
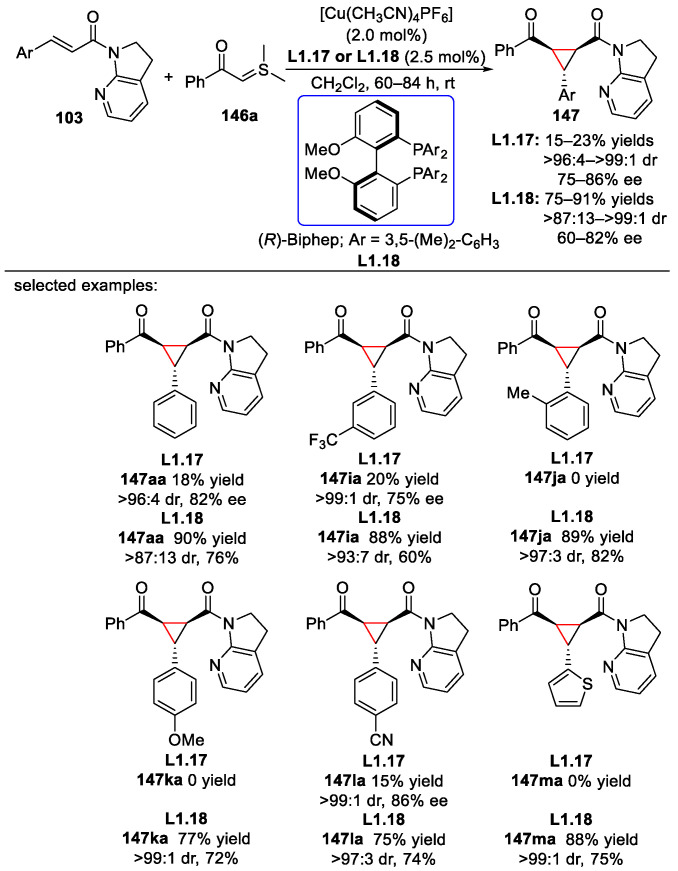
Asymmetric Michael addition-initiated ring-closure reactions of *β*-aryl *α,β*-unsaturated 7-azaindoline amides and sulfur ylides.

The *β*-alkyl *α*,*β*-unsaturated 7-azaindoline amide was also explored under the current catalytic system, and the corresponding products **147aa′**–**147ah′** were easily obtained with good yields and stereoselectivities (Scheme 74). Compared to *β*-alkyl *α*,*β*-unsaturated 7-azaindoline amides, *β*-aryl *α*,*β*-unsaturated 7-azaindoline amides showed lower activity, which required further screening of chiral ligands in the catalytic system. Finally, the authors found a suitable ligand **L1.18** for *β*-aryl *α*,*β*-unsaturated 7-azaindoline amides, although the corresponding product **147a** was only 76% ee. Compared with the catalysis of the Cu/**L1.17** complex, the performance of the Cu/**L1.18** complex was better in the reaction between *β*-aryl *α*,*β*-unsaturated 7-azaindoline amides with different substituents and sulfur ylides, in which the products **147aa**–**147ma** were afforded, with excellent dr values and uniformly increased ee values (Scheme 75).

**Scheme 76:**
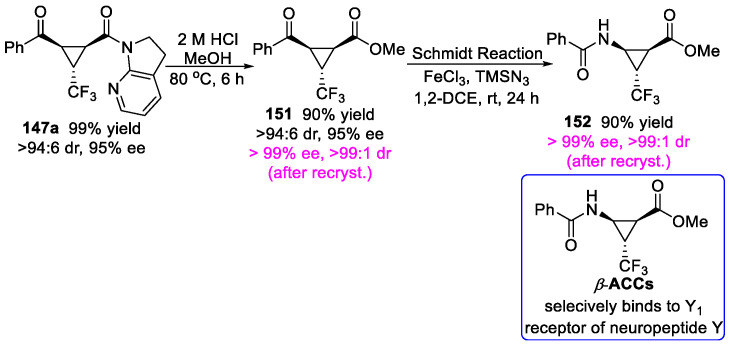
**147a** was converted to **152** via the Schmidt reaction.

To showcase the practicality of 1,2,3-substituted cyclopropanes, the authors conducted further investigations into various transformations of cyclopropanes. It is worth noting that the 7-azindoline group of the cyclopropane derivative **147a** was removed under acidic conditions, and the product ester **151** was afforded with an unchanged dr value. In the presence of the Lewis acid FeCl_3_, the ketone carbonyl of ester **151** underwent a Schmidt rearrangement reaction with TMSN_3_, and the desired product, **152,** showed high regioselectivity (Scheme 76). Product **152** was an important precursor in pharmaceutical chemistry because of its similar structure to the β-aminocyclopropane carboxylic acids (*β*-ACCs), a special synthon of polypeptide ligands with high affinity for neuropeptide Y1 receptors.

**Scheme 77:**
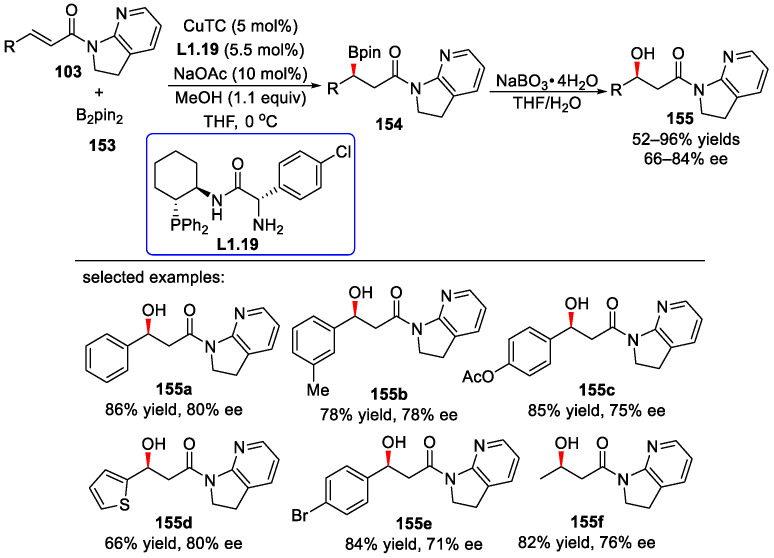
Asymmetric 1,4-hydroboration reactions of *α,β*-unsaturated 7-azaindoline amides and B_2_pin_2_.

**Scheme 78:**
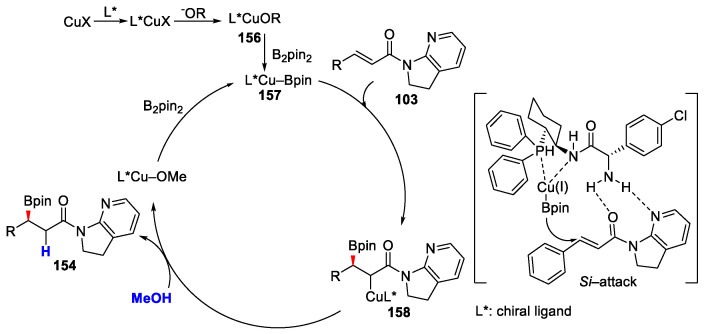
A possible mechanism for 1,4-hydroboration of *α,β*-unsaturated 7-azaindoline amides and B_2_pin_2_.

In 2023, Wu’s group [101] explored an asymmetric 1,4-hydroboration of *α,β*-unsaturated 7-azaindoline amides **103** and B_2_pin_2_ **153** using a Cu(I)/**L1.19** complex as the catalyst. Treatment of alkyl borate esters **154** with NaBO_3_·4H_2_O gave the expected products *β*-hydroxy amides **155** in moderate to good yields with moderate enantioselectivities (up to 96% yield and 84% ee) (Scheme 77). The possible mechanism of the 1,4-hydroboration reaction was proposed, as shown in Scheme 78. First, copper salts interact with chiral ligands **L1.19** and NaOAc to form the L*CuOR complex **156**, and then react with B_2_pin_2_ to provide the active L*Cu-Bpin complex **157**. The *α,β*-unsaturated 7-azaindoline amide **103** is then activated by binding to the hydrogen bond donors of complex **157**. The *Si* face of complex **157** attacks *α,β*-unsaturated 7-azaindoline amide **103**, resulting in the formation of intermediate **158**. In the presence of the proton source MeOH, the intermediate **158** is converted into target product **154**, releasing the species L*CuOMe into the next catalytic cycle.

## 4. Summary and Outlook

As discussed in this review, great progress has been made in metal-catalyzed and organo-catalyzed asymmetric reactions in recent years, using 7-azazindoline amides as universal reagents. The use of various *α*-substituted-7-azaindoline amides and *α,β*-unsaturated 7-azaindoline amides as synthons has greatly expanded the range of substrates for enolization chemistry. This has allowed for the development of new transformations to obtain compounds that are difficult to synthesize through other reactions. In this research area, it is observed that metal catalysts and organic catalysts can be utilized for the catalytic conversion of such reactions as the asymmetric aldol reaction, Mannich reaction, conjugate addition, 1,3-dipole cycloaddition, Michael/aldol cascade reaction, aminomethylation, and Michael addition-initiated ring-closure reaction. However, there relatively few reactions are catalyzed by organocatalysts. One case of the Michael/aldol cascade reaction is reported by Yuan’s group. Therefore, the asymmetric reaction of 7-azaindoline amide, catalyzed by the organocatalyst, has great research potential due to its application in the field of pharmaceutical science. The organocatalysis strategy is currently one of the most thriving research areas in contemporary organic synthesis [102,103,104,105,106]. Moreover, the number of asymmetric reactions with *α*-substituted 7-azaindoline amides and *α,β*-unsaturated 7-azaindoline amides as synthons is still limited, as well as the unabundant types and quantities of 7-azaindoline amides. In other words, developing other types of 7-azaindoline amides and applying them to novel asymmetric reactions will be an interesting but challenging research direction. Further study is needed to investigate the coordination mode between 7-azaindoline amides and metal or organic catalysts, which are possibly verified through theoretical calculations. In general, the relevant examples described in this review highlight the unique utility and potential applications of 7-azaindoline amide as a synthetic precursor for the construction of structurally diverse chiral carboxylic acid compounds through a variety of novel reactions. It is believed that more breakthrough findings on 7-azaindoline amides will be reported in the future.

## Data Availability

Not applicable.
